# BicPAM: Pattern-based biclustering for biomedical data analysis

**DOI:** 10.1186/s13015-014-0027-z

**Published:** 2014-12-16

**Authors:** Rui Henriques, Sara C Madeira

**Affiliations:** INESC-ID and Instituto Superior Técnico, Universidade de Lisboa, Lisbon, Portugal

**Keywords:** Biclustering, Pattern mining, Biomedical data analysis

## Abstract

**Background:**

Biclustering, the discovery of sets of objects with a coherent pattern across a subset of conditions, is a critical task to study a wide-set of biomedical problems, where molecular units or patients are meaningfully related with a set of properties. The challenging combinatorial nature of this task led to the development of approaches with restrictions on the allowed type, number and quality of biclusters. Contrasting, recent biclustering approaches relying on pattern mining methods can exhaustively discover flexible structures of robust biclusters. However, these approaches are only prepared to discover constant biclusters and their underlying contributions remain dispersed.

**Methods:**

The proposed BicPAM biclustering approach integrates existing principles made available by state-of-the-art pattern-based approaches with two new contributions. First, BicPAM is the first efficient attempt to exhaustively mine non-constant types of biclusters, including additive and multiplicative coherencies in the presence or absence of symmetries. Second, BicPAM provides strategies to effectively compose different biclustering structures and to handle arbitrary levels of noise inherent to data and with discretization procedures.

**Results:**

Results show BicPAM’s superiority against its peers and its ability to retrieve unique types of biclusters of interest, to efficiently deliver exhaustive solutions and to successfully recover planted biclusters in datasets with varying levels of missing values and noise. Its application over gene expression data leads to unique solutions with heightened biological relevance.

**Conclusions:**

BicPAM approaches integrate existing disperse efforts towards pattern-based biclustering and provides the first critical strategies to efficiently discover exhaustive solutions of biclusters with shifting, scaling and symmetric assumptions with varying quality and underlying structures. Additionally, BicPAM dynamically adapts its behavior to mine data with different levels of missing values and noise.

## Introduction

Biclustering, a local approach for clustering, seeks to find sub-matrices (biclusters), subsets of rows with a highly correlated expression pattern across a subset of columns. Biclustering has been extensively applied in gene expression data analysis [1], since small groups of genes can participate in multiple cellular processes or pathways of interest that may be only active in a subset of the conditions under analysis. Biclustering has been also applied to group mutations and copy number variations [2], to analyze biological networks [3], and to study translational [4], chemical [5] or nutritional data [6].

Biclustering involves hard combinatorial optimization. In particular, its complexity increases when rows and columns are allowed to participate in more than one bicluster (non-exclusive structure) and in no bicluster at all (non-exhaustive structure). Hence most existing algorithms are either based on greedy or stochastic approaches [1,2,7,8], potentially producing sub-optimal solutions, or on finding a constrained number, structure or type of biclusters [1,2,9].

The state-of-the-art attempts to tackle biclustering using pattern mining techniques allow for exhaustive and flexible searches and show solid levels of efficiency [10,11]. The fact that pattern mining research is driven by scalability requirements [12], opens a critical direction to perform biclustering. Interestingly, the existing pattern-based approaches for biclustering – such as BiModule [13], DeBi [10], RAP [14] and GenMiner [15] – provide complementary principles of interest for this field. However, these principles are not yet integrated. Additionally, existing approaches only discover biclusters with constant profiles [10,13,14], and are not able to handle missing values or medium to high levels of noise. This work aims to target these limitations by proposing a pattern-based biclustering approach, BicPAM, that is able to combine existing potentialities from state-of-the-art pattern-based approaches with two critical novel contributions:

- flexible exhaustive solutions: arbitrary number of (potentially overlapping) biclusters with additive, multiplicative and symmetric assumptions using multiple ranges of values;
- biclustering behavior dynamically adapted to deal with varying levels of noise and missing values.

To our knowledge, this is the first biclustering approach that is able to support and combine each of these two contributions. The importance of these contributions is shown experimentally over synthetic and biological data. Additionally, experimental results on both synthetic and real datasets demonstrate the efficiency and effectiveness of the pattern-based biclustering algorithms proposed in BicPAM.

The paper is organized as follows. *Background* covers essential concepts from biclustering and pattern mining, and surveys the contributions from existing pattern-based biclustering approaches. *BicPAM: pattern-based biclustering* describes the proposed algorithms. In *Results*, we assess BicPAM’s performance on synthetic and real data. Finally, the contributions and implications of this work are synthesized.

## Background

This section introduces fundamental concepts of biclustering and pattern mining, and surveys the related work on pattern-based biclustering.

### **Definition1**.

Given a matrix, *A*=(*X*,*Y*), with a set of rows *X*={*x*_1_,..,*x*_*n*_}, columns *Y*={*y*_1_,..,*y*_*m*_}, and elements $a_{\textit {ij}}\in \mathbb {R}$ relating row *i* and column *j*:

- A *biclusterB*=(*I*,*J*) is a *r*×*s* submatrix of *A*, where *I*=(*i*_1_,..,*i*_*r*_)⊂*X* is a subset of rows and *J*=(*j*_1_,..,*j*_*s*_)⊂*Y* is a subset of columns;
- The **biclustering task** is to identify a set of biclusters $\mathcal {B}=\{B_{1},..,B_{p}\}$ such that each bicluster *B*_*k*_=(*I*_*k*_,*J*_*k*_) satisfies specific *criteria of homogeneity*, where *I*_*k*_⊂*X*, *J*_*k*_⊂*Y*, and $k\in \mathbb {N}$.

Approaches to solve the biclustering task either explicitly or implicitly rely on a merit function to define the homogeneity criteria. An illustrative function is the variance of bicluster’s values. Merit functions either guarantee intra-bicluster homogeneity, the overall homogeneity of the output set of biclusters (inter-bicluster homogeneity), or both. When combined within specific search procedures, merit functions are to define the type, quality and structure of biclustering solutions [1].

Merit functions can be defined to locally maximize greedy iterative searches [7,8,16–19], to combine row- and column-based clusters [20–22], to exploit matrices recursively [23], and to stochastically model the target solution [6,24]. In exhaustive searches, which commonly rely on constrained formulations, merit functions are the heuristics that guide the space exploration [9,25].

**Figure 1:**
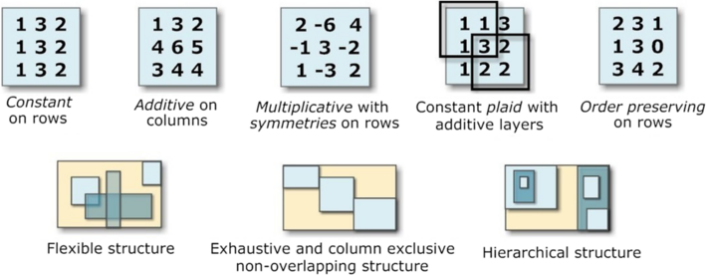
**Illustrative bicluster types and biclustering structures.**

Figure 1 presents different types and structures of biclusters. Biclusters can follow constant or more flexible models, with coherency on rows or columns [1]. Biclusters under an additive-multiplicative model, also referred as shifting-scaling biclusters, can be discovered using merit functions based on *δ*-offsets of noise [17,25], on vector-angle cosines [21], or on generative models of linear dependencies [2]. Biclusters with symmetries can be discovered by differential biclustering methods [9,26] and by few others [14]. Additionally, plaid [6] and order-preserving [19] types of biclusters have also been tackled [27,28]. Multiple biclustering structures have been proposed [1], with some approaches constraining them to exhaustive, exclusive, non-overlapping structures, and others allowing more flexible structures with arbitrarily positioned overlapping biclusters [7].

### Pattern mining

Patterns are itemsets, rules or substructures that appear in a dataset with frequency no less than a specified threshold. Finding patterns is critical to derive relations from data.

#### **Definition2**.

Let  be a finite set of items, and *P* be an itemset $P\subseteq \mathcal {L}$. A *transactiont* is a pair (*t*_*id*_,*P*) with $id\in \mathbb {N}$. An *itemset databaseD* over  is a finite set of transactions {*t*_1_,..,*t*_*n*_}.

#### **Definition3**.

A transaction (*t*_*id*_,*P*) contains *P*^′^, denoted *P*^′^⊆(*t*_*id*_,*P*), if *P*^′^⊆*P*. The *coverageΦ*_*P*_ of an itemset *P* is the set of all transactions in D in which the itemset *P* occurs: *Φ*_*P*_={*t*∈*D*∣*P*⊆*t*}. The *support* of an itemset *P* in *D*, denoted *sup*_*P*_, can either be absolute, being its coverage size |*Φ*_*P*_|, or a relative threshold given by |*Φ*_*P*_|/|*D*|.

#### **Definition4**.

Given an itemset database *D* and a minimum support threshold *θ*, the *frequent itemset mining* (FIM) problem consists of computing the set $\{P \mid P \subseteq \mathcal {L}, {sup}_{P} \geq \theta \}$.

A *frequent itemset* is an itemset with *sup*_*P*_≥*θ*. An accepted pattern is a frequent itemset that satisfies any other placed constraints over *D*.

To illustrate these concepts, consider the following itemset database, D _*ex*_= {(t _1_,{B,E,G}), (t _2_, {A,B,C,E,H,J}), (t _3_,{A,B,D,H,J}), (t _4_,{D,H,J}), (t _5_,{A,H,J}), (t _6_,{A,G})}. We have $|\mathcal {L}|=|\{A,..,J\}|=10, \Phi _{\{B,J\}}=\{t_{2}, t_{3}\}$ and *sup*_{*B*,*J*}_=|{*t*_2_,*t*_3_}|/6=0.(3). For *θ*=4, FIM tasks returns {{*A*},{*H*},{*J*},{*H*,*J*}}.

Since FIM proposal [29], multiple extensions have been proposed, ranging from scalable data mining methodologies to multiple condensed and approximated pattern representations.

#### **Definition5**.

Given an itemset matrix, a support threshold *θ*, and the coverage function $\Phi : 2^{\mathcal {L}}\rightarrow 2^{D}$ that maps an itemset *P* to its set of supporting transactions:

- A frequent itemset *P* is an itemset that satisfies |*Φ*(*P*)|≥*θ*;
- A *closed* frequent itemset is a frequent itemset with no superset with same support $\phantom {\dot {i}\!}\left (\forall _{P'\supset P}|P'|<|P|\right)$;
- A *maximal* frequent itemset is a frequent itemset with all supersents being infrequent, $\forall _{P' \supset P}|\Phi (P')|< \theta $.

A frequent itemset is maximal if all its supersets are infrequent, while it is closed if it is not a subset of an itemset with the same support. Considering the previously introduced itemset database D _*ex*_, a given threshold *θ*= 3 and |*P*|≥2, there is one maximal frequent itemset ({*A*,*H*,*J*}) and there are two closed frequent itemsets ({*A*,*H*,*J*} and {*H*,*J*}).

#### **Definition6**.

Consider two itemsets $P\in 2^{\mathcal {L}}$ and $P'\in 2^{\mathcal {L}}$, where *P*^′^⊆*P*, and a predicate *M*. *M* is *monotonic* when *M*(*P*)⇒*M*(*P*^′^) and *M* is *anti-monotonic* when ¬*M*(*P*^′^)⇒¬*M*(*P*).

These properties are the basis of FIM, either for candidate generation or pattern growth methods, with horizontal or vertical data formats.

### Pattern-based biclustering

The homogeneity criteria (Definition 1) in pattern-based approaches for biclustering is obtained through support and confidence-correlation metrics. Pattern-based approaches allow for an efficient and exhaustive space search that produces an arbitrary high number of biclusters within a flexible structure.

#### **Definition7**.

Given a matrix *A* and a minimum support threshold *θ*, a set of *biclusters* ∪_*k*_*B*_*k*_, where *B*_*k*_=(*I*_*k*_,*J*_*k*_), can be derived from the set of frequent itemsets ∪_*k*_*P*_*k*_ by either mapping $\phantom {\dot {i}\!}(I_{k},J_{k})=(\Phi _{P_{k}},P_{k})$ to compose biclusters with coherency on rows, or by mapping $\phantom {\dot {i}\!}(I_{k},J_{k})=(P_{k},\Phi _{P_{k}})$ to compose biclusters with coherency on columns.

**Figure 2:**
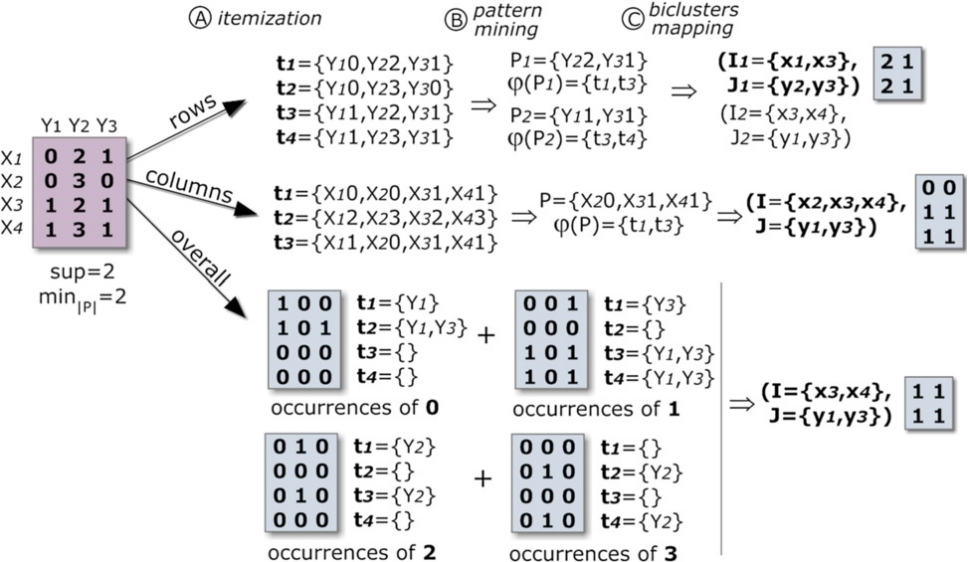
**Discovering biclusters with a constant assumption across rows (a), columns (b) and overall elements (c) using frequent itemset mining.** Column identifiers (y _1_, y _2_, y _3_) are combined with the observed values {0,1,2,3}, and FIM applied under a parameterizable support threshold (*θ*=2∧|*P*|≥2). Constant values on columns can be mined using the transpose matrix. To find biclusters with constant values overall, each item needs to be separately mined.

A pattern-based approach to biclustering relies on an itemization step, where the original matrix is transformed into an itemset database, followed by the application of FIM methods under a low support threshold. For real-value matrices, normalization and discretization procedures are applied. Then, the discrete value of each cell is concatenated with the respective column index. Each transaction of the target itemset database corresponds to a row with these new values. FIM is applied over the database to mine frequent patterns, which are then used to derive biclusters with constant values on rows. Constant values on columns can be mined using the transpose matrix. To find a more constrained type of biclusters, such as constant values overall, each item needs to be mined separately. Figure 2 illustrates how to deliver such types of biclusters using frequent patterns.

Although the state-of-the-art pattern-based biclustering methods follow this general behavior, they have varying structural specificities that affect both the efficiency and the quality of the output. Two classes of PM-based biclustering approaches can be considered: approaches that directly apply pattern miners over discrete matrices, and approaches that target numeric matrices by customizing the support metric. To our knowledge, BiModule [13], DeBi [10], Bellay’s et al. [30] and GenMiner [15] are the state-of-the-art methods for this first class of PM-based biclustering. BiModule [11,13] allows for a parameterized multi-value itemization of the input matrix to discover constant biclusters derived from (closed) frequent patterns using the LCM miner [31]. DeBi [10] derives biclusters from (maximal) frequent patterns mined over binarized matrices using the MAFIA miner [32], and places key post-processing principles to adjust biclusters in order to guarantee their statistical significance. Bellay’s et al. [30] use the Apriori miner with additional principles to evaluate the functional coherency of the discovered biclusters against the background noise. GenMiner [15] includes external knowledge within the input matrix to derive biclusters from association rules that relate annotations (external grouping of rows or columns) with computed clusters of rows and columns from (closed) frequent patterns using CLOSE [33]. Although other biclustering approaches seize contributions from these previous works [34,35], we do not refer to them as PM-based appproaches if the core mining task does not rely on FIM.

The itemization step is optional for the second class of methods [36]. To our knowledge, RAP [14], RCB discovery [36] and ET-bicluster [37] are the state-of-the-art methods in this context. RAP [14] plugs an adapted range-based metric to mine constant biclusters on rows (or columns), while RCB discovery targets biclusters with constant values overall [36]. ET-bicluster extends the previous approaches to discover noisy biclusters, although an exhaustive enumeration of biclusters is not guaranteed [37]. Alternative support metrics with dedicated Apriori-based searches have been additionally referred in literature [38–40].

## BicPAM: pattern-based biclustering

**Figure 3:**
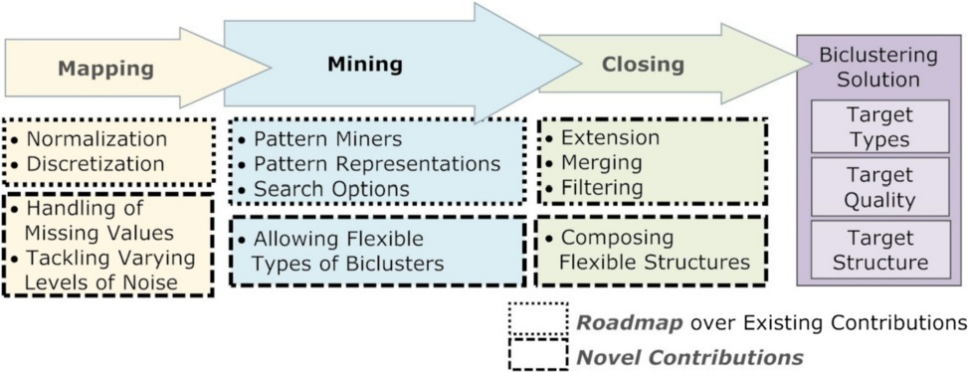
**BicPAM’s methodology.** BicPAM relies on three steps that determine the type, quality and structure of the biclustering solutions. Within each step, we make available principles based on existing contributions. Additionally, we propose key strategies within each step for the handling of noise, the accommodation of more flexible types of biclusters (with additive, multiplicative and symmetric properties) and the composition of alternative structures of biclusters.

The proposed pattern-based biclustering approaches (BicPAM) are an ordered composition of the three-stage: *mapping*, *mining* (pattern discovery), and *closing* (or post-processing) steps. BicPAM relies on both existing and novel principles for each step. The core step is the *mining* step, corresponding to the application of the target pattern miners. This step is driven by the considered pattern discovery approach, target patterns and search properties. The *mapping* step consists in the itemization of a real-value matrix into an itemset matrix. This step is driven by normalization and discretization criteria and it may use different principles to handle outlier, numeric or missing elements. Finally, the *closing* step consists on the post-processing of the output patterns to affect the structure and quality of the target biclusters. Figure 3 clarifies how BicPAM relies on the existing pattern-based contributions and pinpoints the novel principles proposed for each step.

The homogeneity criteria can be intentionally defined to search for specific types and structures of biclusters and to affect their quality. The bicluster *type* depends on the allowed coherency patterns and on their orientation (row, column or overall), the solution *structure* depends on the number, size and positioning of biclusters, and, finally, the *quality* defines the allowed noise associated with a single bicluster or with a set of biclusters.

BicPAM is introduced in three following sections. First, we describe the core steps of BicPAM (*BicPAM outline*). Second, we go further and incorporate new methods to deal with missing values and arbitrary high input levels of noise (*Affecting the quality of pattern-based biclusters*). Finally, we propose further algorithmic solutions for the discovery of biclusters allowing symmetries and following additive and multiplicative assumptions (*Allowing more flexible types of biclusters*).

### BicPAM outline

This section describes the structural behavior of BicPAM by surveying principles for the mining, mapping and closing steps. These principles are either derived from the existing pattern-based approaches for biclustering or from advances in the pattern mining field.

#### Mining step

Understandably, non-constrained settings, where the number of biclusters and their properties is not known apriori, require efficient searches. Pattern mining approaches have been tuned during the last decades to be computationally efficient. Therefore, their adequate use for biclustering is critical and depends essentially on three points described below: *1)* the adopted pattern-based approach to biclustering, *2)* the target pattern representation, and *3)* the search strategy.

*1) Pattern-based Approach*

##### **Definition8**.

Let  be a set of ordinal items, a bicluster is a sub-matrix (*I*,*J*)⊆*A* with its elements $a_{\textit {ij}}\in \mathcal {L}$ defining a pattern profile. A constant bicluster can follow: *i)* an *overall constant assumption* where *a*_*ij*_=*c* and $c\in \mathcal {L}$, *ii)* a *column-based constant assumption* where *a*_*ij*_=*c*_*j*_ and $c_{j}\in \mathcal {L}$, or *iii)* a *row-based constant assumption* where *a*_*ij*_=*c*_*i*_ and $c_{i}\in \mathcal {L}$.

Pattern-based biclustering under a constant assumption is the ordinary case. DeBi [10], BiModule [13] or GenMiner [15] only target this type of biclusters. These approaches either rely on Frequent Itemset Mining (FIM) or on association rules, which contrasts with traditional approaches [9,18]. The support threshold defines the minimum number of rows in a bicluster. In the context of gene expression, a low support is critical since high expression coherency is only observed for small groups of genes and conditions. Additionally, a post-pruning to the frequent itemsets can be performed in order to filter frequent itemsets below a minimum number of columns and above a maximum number of rows and columns.

From the point of view of an itemized database, the FIM-based biclusters are perfect biclusters, that is, they do not allow value-variations in any of its elements. Contrasting, from the point of view of the input real-value matrix, these biclusters can handle noise since two elements with the same item may be numerically distant. The number of items can be used to control the noise-tolerance. However, regardless of the number of items, a common drawback appears when two elements have similar real-values but different items assigned. We refer to this drawback as the items-boundary problem.

BiModule [11] and DeBi [10] are representative FIM-based biclustering approaches. Since their running time is comparable to greedy algorithms, they offer a simplistic way to deal with noise and overlapping structures [13]. However, the items-boundary problem can lead to the partitioning of large biclusters into smaller ones (with many being filtered as they no longer satisfy the support criterion).

In order to mine frequent itemsets with different properties, the notion of support of an itemset can be redefined. RAP [14] uses a customized anti-monotonic range support merit function. A FIM-based algorithm is used to discover range patterns from real-value matrices without the need for discretization. However, efficiency is strongly penalized.

An additional option to pattern-based biclustering is to derive biclusters from association rules. An association rule, an implication between two itemsets, can affect the properties of the corresponding bicluster as it constrains the levels of confidence among rows. Optionally, correlation metrics can be adopted to augment the confidence-support metrics with new interestingness criteria. GenMiner [15] uses association rules to compose biclusters. However, the adoption of association rules is only preferred over FIM-based approaches when knowledge regarding the dependencies across rows (or columns) is available.

BicPAM uses frequent itemsets as the default pattern-based option to biclustering. Range-based approaches are only selected for small-to-medium datasets. Finally, in the presence of domain knowledge (such as functional groups of genes or dependencies on conditions), BicPAM relies on association rules to compose biclustering solutions.

*2) Pattern Representations*

The target pattern representation depends essentially on: *1)* the selected type and structure of biclusters, and *2)* the post-processing needs. Efficiency is not a strong criterion, since only subtle gains are observed for methods that target constrained representations, such as closed and maximal representations.

The use of all frequent itemsets leads to biclustering solutions with a high number of (potentially redundant) biclusters (if contained by another bicluster), which can degrade the performance of the mining and closing steps. Contrasting, the use of maximal itemsets leads to biclusters with the columns’ size maximized. Maximal itemsets for biclustering are adopted in DeBi [10]. Such flattened biclusters are particularly interesting when there is an extension step to be performed to include new rows for the discovered biclusters. However, since both vertical and smaller biclusters are avoided, maximal-based biclusters lead to incomplete solutions as they are just a subset of all valid biclusters.

Finally, by using closed itemsets, we allow for overlapping biclusters only if a reduction on the number of columns from a specific bicluster results in a higher number of rows. Note that to obtain *maximal biclusters* – biclusters that cannot be extended without the need of removing rows and columns – closed patterns need to be used instead of maximal patterns. FIM-based BiModule [13] and rule-based GenMiner [15] use closed itemsets as the means to compose biclusters.

**Figure 4:**
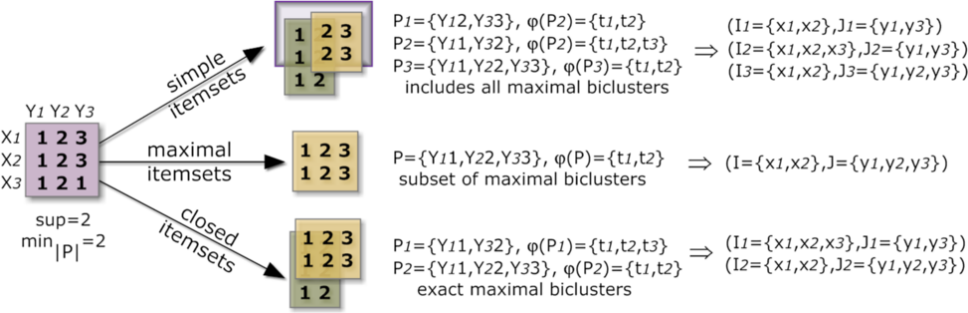
**Comparison of biclustering solutions using frequent itemsets, maximal frequent itemsets and closed frequent itemsets.**

BicPAM uses frequent closed patterns as the default representation. The set of all and maximal frequent patterns are also made available in BicPAM. An illustration on how different types of pattern representations lead to structurally different biclustering solutions is provided in Figure 4.

*3) Search Strategies*

The definition of the search setting depends essentially on: *1)* the fit of the search with the target biclustering task, and *2)* the chosen implementation.

The choice on whether to use Apriori-based [41], pattern-growth [42] or combined approaches [43], mainly depends on the dataset density and fixed support thresholds. Dense matrices under low support thresholds benefit from pattern-growth or combined methods. The choice on whether to use a mining method that has a vertical versus an horizontal data format [43] depends essentially on the type of biclusters we are targeting. If we want to find constant values across rows or on both dimensions, we usually benefit from using searches over horizontal data formats [35]. This is particularly true for most GE matrices where the total number of genes largely exceeds the total number of conditions. If we want to find constant values across columns (when *n*>*m*), a vertical data format should be the choice, as the performance searches based horizontal formats exponentially degrades with an increasing number of items.

Several algorithms were developed for each of these search strategies. However, their properties should be carefully assessed, as their nature is most of the times optimized towards specific sets of datasets. In the DeBi [10], BiModule [11] and GenMiner [15] biclustering tasks, Mafia [32], LCM [31] and CLOSE [33] are, respectively, the algorithmic choices.

BicPAM makes available a variant of FP-Growth that traces the set of transactions per frequent pattern [44] (default option), Charm [45], AprioriTID [41] and Eclat [43]. Finally, Carpenter [46] and Cobbler [47] are additional algorithmic choices in BicPAM to compose biclusters with a large number of columns and for large-scale datasets.

#### Mapping step

*Normalization* techniques are often required to enhance differences across rows and/or columns. Alternative methods have been reported [34,48]. BicPAM allows the normalization criteria to be applied in the context of a row, a column or the overall matrix. Additionally, it makes available a zero-mean value to allow for symmetries and to provide a simple setting for the approximation of probabilistic distributions. In the presence of missing and outlier elements, a masking bitmap can be used in order to exclude them from the computation of the mean and dispersion metrics.

*Discretization* is an additional key step for pattern-based methods relying on itemset databases. Although discretization may imply loss of information, it alleviates the noise dilemma [26] and it is the cost to pay for exhaustive searches. BicPAM makes available multiple discretization options with key implications on the target solution. Two axes are considered: *1)* the number of items (also referred as symbols) and *2)* the target method to map the normalized real-value matrix into a itemset database. Increasing the number of items is commonly used to improve quality, but it reduces the average size of biclusters and the number of biclusters produced. A sensitivity analysis on the impact of choosing different number of items was performed in Bidens [34] and BiModule [13].

**Figure 5:**
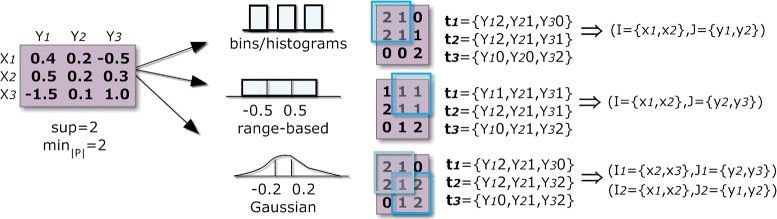
**Impact of discretization options available in BicPAM.**

The three discretization methods made available in BicPAM are illustrated in Figure 5. The use of fixed ranges (potentially equal sized intervals between the observed maximum and minimum) is the simplest discretization option, but commonly leads to an accentuated weak distribution of items and is prone to the items-boundary problem. The first problem can be corrected using a percentage-based method for the depth partitioning of items that leads to intervals containing approximately the same number of items. Bidens [34] uses this equal-depth partitioning method over a data context where outliers are temporarily removed. Finally, alternative distributions can be used to combine the properties of the previous solutions, such as the setting proposed in Nordi [15]. By finding multiple suitable curves (for each row or column) or one suitable overall curve for approximating the matrix, we can either use threshold methods or directly compute the statistical cutoff points to create equally-distributed areas. In the illustration, a Gaussian distribution is selected to minimize the loss of potentially relevant biclusters.

#### Closing step

Similarly to mining and mapping options, post-processing criteria can be used to minimize the two challenges of the noise dilemma. One challenge results from a too restrictive noise tolerance, commonly associated with considering a high number of items, which leads to many small sized biclusters. The other challenge is related with heightened levels of noise allowance, commonly appearing in binarized partitions and under a relaxed levels of support or confidence. To handle these challenges and to treat the problem of the explosion of valid biclusters (commonly connected with overlapping biclusters), BicPAM enables the use of criteria structured according to three stages: *1)* extension, *2)* merging and *3)* filtering.

*1) Extension Options*

Three optional and non-exclusive strategies can be used to extend the discovered biclusters such that the resulting solution still satisfies some pre-defined homogeneity criteria. First strategy consists on the use of statistical tests to include rows or columns over each bicluster as proposed in DeBi [10]. Second strategy relies on traditional approaches and on their merit functions for further extensions as long as the solution satisfies either the intra- or inter-bicluster homogeneity criteria. Finally, we propose a third strategy that uses patterns discovered under more relaxed criteria (such as lower support-confidence thresholds) to guide the extension step. When considering lower supports, new columns and rows can be added to the original frequent patterns. Similarly, more relaxed association rules, with less restrictive ways to group the antecedent-consequent, can be used to guide the extension step. The use of simple thresholds, of statistical tests or of merit functions to verify if the bicluster is valid can either be computed using the discretized matrix (item matchings) or, more interestingly, the distances from the original real-value matrix.

*2) Merging Options*

Merging operations serve two goals: noise allowance and overall biclustering structure manipulation. The first goal is driven by the observation that when two biclusters share a significant area it is probable that their merging composes a larger bicluster still respecting some homogeneity criteria. Commonly, such decomposition is related with the items-boundary problem or with a missing value. The simplest criterion to allow the merging is either to rely on the overlapping area (as a percentage of the smaller bicluster), to compute the overall noisy percentage after the merging, or to use advanced homogeneity criteria (potentially relying on the real-values provided by the input matrix). State-of-the-art procedures to efficiently merge pattern-based biclusters include [49,50].

*3) Filtering Options*

Filtering is possible at two levels: *1)* at the bicluster level, and *2)* at the row-column level. The first type of filtering is required to remove duplicates and biclusters that are contained in larger biclusters. The existence of biclusters included in larger biclusters is a necessary result of the extension-merging options and it is a common problem when adopting mining approaches that do not rely on condensed pattern representations. Both DeBi [10] and BiModule [13] provide alternative heuristics to efficiently perform this type of filtering.

The second type of filtering can be used to exclude rows or columns from a particular bicluster in order to intensify its homogeneity. This is usually the case when a low number of items is considered, leading to highly noise-tolerant biclusters. For this purpose, similarly to extension options, we can rely on three strategies: *1)* use statistical tests on each row and column of a particular bicluster in order to identify removals, *2)* rely on existing greedy-iterative approaches and maximize their merit functions (which can imply a reduction on the size of biclusters), and *3)* discover patterns under more restrictive conditions (as higher support and confidence thresholds).

### Affecting the quality of pattern-based biclusters

BicPAM options with impact on the solution quality include:

- Mining step options, including the approach, the support-confidence thresholds, and the pattern representations;
- Mapping step options, including the number of items and the normalization-discretization techniques;
- Closing step options, including the selected extension, merging and filtering approaches, and their criteria thresholds (percentage of noise, overlapping degree, statistical significance levels).

Below, we describe new strategies that BicPAM makes available to handle varying levels of missing values and input noise, and to compose multiple structures of biclusters.

#### Handling missing values

The input matrices can have an arbitrary high number of missing values, as it is common in GE matrices. A non-treated missing value may result in the loss of a critical row and of a column across one or more biclusters. Three different strategies can be applied to treat missing values: *1)* removal, *2)* replacement, and *3)* handling as a special value. The simplest method is to remove the containing row or column (usually the dimension with smaller size).

**Figure 6:**
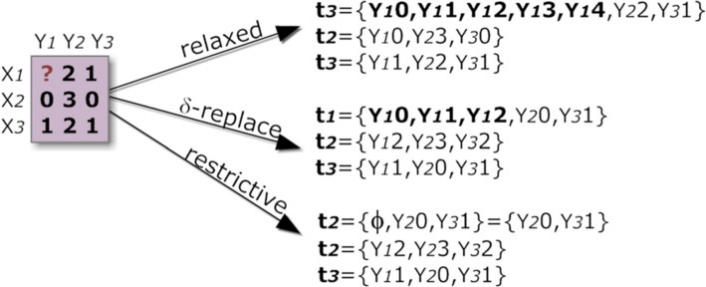
**Mapping methods to handle missings: relaxed, conservative (** ***δ-replace*** **) and restrictive alternatives to imputation.**

Many hole-replacing methods have been proposed [51–53], alleviating the referred problem, although introducing additional noise that can significantly decrease the homogeneity of the output biclusters. For this reason, we propose the use of an additional item that is specially handled according to a level of relaxation handled by the user, as illustrated in Figure 6. The lowest constrained setting (*relaxed*) replaces the missing item by all other adopted items, which again results in transactions with varying size. The medium constrained setting (*δ-replace*) considers multiple items around its value-estimation. If the difference between the estimated value and the centroid-value of a discretization range is less than *δ*, then the item assigned to the range is added. In BicPAM, the default imputation method is based on the mean values for the four nearest neighbor rows. BicPAM default *δ* distances guarantee a lower bound of two items and an upper bound of three items. The highest constrained setting (*restrictive*) removes missing items.

#### Handling varying levels of noise

**Figure 7:**
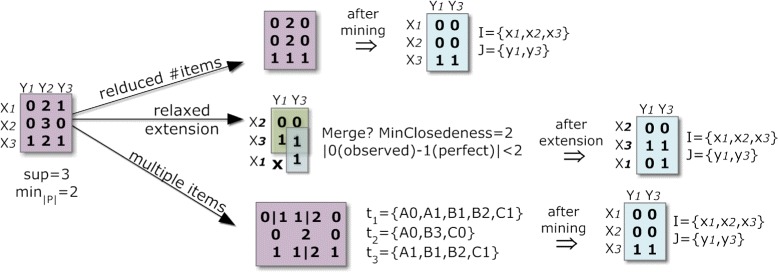
**Strategies to deal with noise-relaxations.**

A key direction to pattern-based biclustering is to consider multiple levels of noise by following one of the three strategies illustrated in Figure 7. *First* strategy (reduced number of items) hierarchically joins contiguous items (items are viewed as being ordinal and no longer nominal) to mine biclusters over matrices with different number of items. Optimizations to this strategy can be made by collapsing items only for some critical areas of the matrix where the presence of biclusters is scarcer. Understandably, the level of noise should be maintained by each bicluster, so that closing steps can be adapted in respect to the quality of the target bicluster. *Second* strategy (relaxed-to-restricted extensions) considers varying levels of noise only after the mining. For instance, the merging of constant biclusters can follow a statistical test sensitive to the closeness of different items (heuristics based on overlapping rows-and-columns should also be considered). *Third* strategy (multiple items) associates one or more items to each element based on a parameterized threshold. Different criteria can be defined to assign a varying number of items per element *a*_*ij*_. Each element can be mapped into two-to-three items based on the distance to their centroids leading to transactions with multiple sizes.

#### Producing alternative biclustering structures

Since the number of biclusters is neither fixed nor depends on the satisfaction of local coverage criterion, pattern-based approaches provide a heightened flexibility for the composition of different biclustering structures. A pattern-based solution is non-exhaustive, non-exclusive and allows overlaps. The task of composing different structures has been poorly addressed in literature, and rather seen as the byproduct of biclustering methods [1]. Below, we introduce a set of principles to compose multiple structures made available in BicPAM.

For an *exhaustive* structure (either overall, across rows or across columns), biclusters can be incrementally merged following, for instance, an hierarchical criteria based on the proximity and the area of biclusters, until all the matrix is covered. If the goal is an *exclusive* structure (either overall, across rows or across columns), a simple strategy is to merge biclusters in order to reduce overlapping across one or both dimensions and, additionally, to filter biclusters that share rows or columns following an relevance criterion (as size or noise level) until exclusivity is guaranteed. Closing options can be specifically used to produce other alternative structures with sharp usability (no need to change the core tasks of pattern-based approaches).

### Allowing more flexible types of biclusters

Below, we extend BicPAM to consider more flexible expression patterns: additive, multiplicative and symmetric coherency.

#### Coherency under additive-multiplicative assumption

##### **Definition9**.

A bicluster (*I*,*J*) follows an *additive model* if *a*_*ij*_=*c*+*α*_*i*_+*β*_*j*_+*η*_*ij*_, where *c* is the typical value within the bicluster, *α*_*i*_ is the adjustment for row *i*∈*I*, *β*_*j*_ is the adjustment for column *j*∈*J* and *η*_*ij*_ is the noise associated with the element. A bicluster (*I*,*J*) follows a *multiplicative model* if $a_{\textit {ij}}=c^{\prime } \times \alpha ^{\prime }_{i}\times \beta ^{\prime }_{j}+\eta _{\textit {ij}}$, which is equivalent to the additive model when *c*=*logc*^′^, $\alpha _{i}=log\alpha ^{\prime }_{i}$ and $\beta _{j}=log\beta ^{\prime }_{j}$.

We propose two pattern-based strategies for the discovery of biclusters with non-constant models of coherency. The first strategy is to use local normalization procedures to correct row- or column-based differences and then to map the task into the search for constant biclusters.

**Figure 8:**
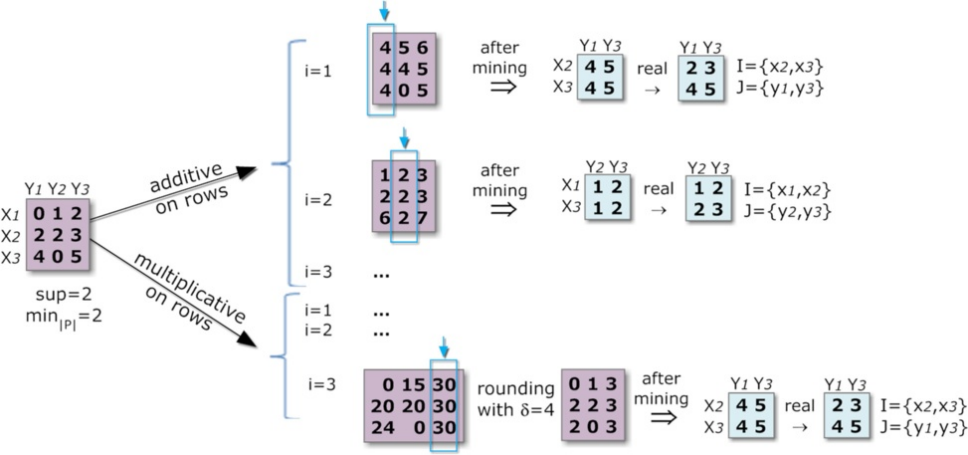
**Pattern-based discovery of biclusters under additive and multiplicative assumptions.**

The second strategy, the default BicPAM option, is to iteratively perform alignments on each column (or row). This guarantees that all the alignments needed to compose these biclusters are considered. Therefore, the selected pattern miner is applied either *m* (or *n*) times, leading to a higher computational complexity. Figure 8 illustrates this strategy.

An additive alignment over a target column *y*_*j*_ can be computed by adding for each element on the row *x*_*i*_ the difference between the maximum of the column and the discretized value *max*(*y*_*j*_)−*a*_*ij*_. A multiplicative alignment over a target column *y*_*j*_ can be computed by adding, for each element on the row *x*_*i*_, the least common multiple between the maximum of the column and the discretized value *lcm*(*max*(*y*_*j*_),*a*_*ij*_). The resulting number of items under an additive assumption is in the worst case the double of the number of items initially considered. The number of final items under a multiplicative model is the size of the *lcm* combinations across the initial items. As illustrated in Figure 8, a distance-based *δ*-error can be considered to gather close items in the multiplicative model due to the lower probability of finding coherent biclusters as a consequence of the resulting large number of items.

#### Coherency under symmetrical assumption

A critical, but less studied, type of biclusters is biclusters with coherent values under symmetrical assumption, also referred as biclusters with sign-changes in literature [1]. Two rows or columns from a bicluster allowing symmetries may have similar absolute values differing in sign. Such biclusters can simultaneously capture activation and repression mechanisms within a biological process.

##### **Definition10**.

A bicluster (*I*,*J*) following a *symmetric model* has either: *i)* symmetries on rows $\hat {a}_{\textit {ij}}=c_{i}\times a_{\textit {ij}}$, where *c*_*i*_∈{−1,1} is the symmetry factor for each row of the bicluster and $a_{\textit {ij}}\in \mathbb {R}$ is a bicluster element defined according to a constant, additive or multiplicative model, or *ii)* on columns $\hat {a}_{\textit {ij}}=c_{j}\times a_{\textit {ij}}$, where *c*_*j*_∈{−1,1} is the column symmetry factor and $a_{\textit {ij}}\in \mathbb {R}$ is an element of a bicluster with coherent values.

For the purpose of finding biclusters with symmetries, the normalization should satisfy the zero-mean criterion. Additionally, if the number of considered items is odd, there is one item being its own symmetric that must be specially handled.

**Figure 9:**
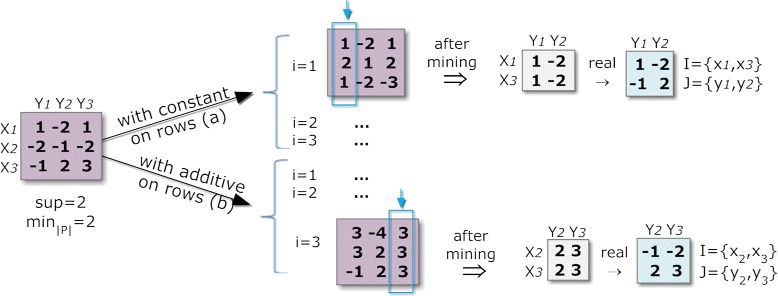
**Pattern-based discovery of biclusters with symmetries for a constant coherency (a) and non-constant coherency (b).**

One option is to align the sign of activity of each row (or column) in order to guarantee consistency of signals for a target column (or row). The top example in Figure 9 illustrates this strategy. An iterative mapping for every column (or row) is possible, although additional efficiency can be achieved by stopping the search when all the sign combinations have been achieved. Nevertheless, the worst case requires the application of a pattern miner *m* times (or *n* times). Note that filtering is a critical step needed to remove potential duplicates resulting from repetitions of alignments for subsets of rows (or columns).

The combination of this strategy with the search for biclusters under an additive or multiplicative model can be expensive (*m*×*m* times iterations). Therefore, BicPAM makes available an additional option to combine the use of the sign and of the additive or multiplicative adjustments together for every column (or row). This model (combined sign and coherent model) is not equivalent to the previous model (sign plus coherent model), since it assumes that additions or multiplications are not absolute but depend on the activity slope sign. Here, the value adjustment of a particular element is also affected by the sign, which can lead to an additional number of items. This strategy is illustrated in the bottom example of Figure 9.

### BicPAM algorithm and complexity analysis

The algorithmic basis of BicPAM is described in Algorithm 1. Although BicPAM follows a plug-and-play style, default and data-driven parameterizations are made available. In particular, *lines 40-44* and *37* describe BicPAM behavior in the absence of user-driven parameterizations. This is performed by either relying on estimation procedures or on convergence criteria based on thresholds such as the relative area covered by biclusters or the minimum number of biclusters.



The computational complexity of BicPAM is bounded by the pattern mining task and computation of similarities among biclusters. For this analysis, we cover major computational bottlenecks related with each one of the three major steps of BicPAM. Within the *mapping* step: outlier detection, normalization, discretization, and noise correction procedures (such as the assignment of multiple items) are linear on the size of the matrix, *Θ*(*nm*). The optional distribution fitting tests and parameter estimations to dynamically select an adequate discretization procedure are also *Θ*(*nm*). These tests and estimations rely on the calculation of approximated statistical ratios [54]. Handling missings by removing the respective element or by replacing them by a special dedicated item is also *Θ*(*nm*). However, when an imputation method is selected, the complexity is upper bounded by *Θ*(*hnm*), where *h* is the number of missing values. In BicPAM implementation, the nearest neighbor rows and columns are computed for the estimation of each missing value.

The cost of the *mining step* depends on two factors: the complexity of the pattern miner and the need for iterations for the discovery of non-constant profiles. The cost of the pattern mining task depends essentially on: the number and size of transactions (*γnm*, where *γ*≥1 captures the increase in size related with noise and missings handlers), the frequency distribution of items ($\{\mathcal {L}\times Y\}\rightarrow \mathbb {N}$), the minimum support *θ*, the pattern representation and the selected mining procedure. A detailed analysis of this complexity has been attempted in literature [55] and it is out of the scope of this paper. The reader should also keep in mind that there has been proposals to guarantee the scalability of pattern miners recurring to partitioning and approximation methods [12]. Let $\Theta (\wp (\gamma,n,m,|\mathcal {L}|,\theta))$, or simply *Θ*(*℘*), to be the complexity of a pattern mining task. When there is the need for the iterative application of the core mining procedure, the overall search is bounded by *Θ*(*d*×*℘*), where $d=min\left ({n \choose 2},m\right)$ when allowing symmetries, $d=min\left ({n \choose |\mathcal {L}|},m\right)$ when allowing shifts, and $d=min\!\left ({n \choose \sharp lcm},m\right)$ when allowing scaling factors.

The cost of the *closing* step depends essentially on two factors: the complexity of computing similarities among biclusters (required for merging and filtering biclusters) and the complexity of extending and reducing biclusters. To compute similarities a tree structure is created where each node represents a gene and each leaf corresponds to a bicluster. Only biclusters sharing a branch over a threshold based on the input overlapping degree are candidates for merging and filtering. Filtering a bicluster results in the removal of its leaf and dedicated nodes. Merging two biclusters results on the combination of the target branches. These tasks have an average complexity of $\Theta \!\left ({k \choose k/2}\bar {r}\bar {s}\right)$, where *k* is the number of biclusters and $\bar {r}\bar {s}$ their average size. Extending biclusters relies on quick tests based on the coherency of each new column or row and therefore the complexity of this task is respectively $\Theta (k'\bar {r}m)$ or $\Theta (k'n\bar {s})$, where *k*^′^ is the number of biclusters after merging and filtering. Removing rows or columns from biclusters is $\Theta (k'\bar {r}\bar {s})$.

In this context, the complexity of BicPAM is bounded by $\Theta \!\left (hnm+d\wp +{k \choose k/2}\bar {r}\bar {s}+k'(\bar {r}m+n\bar {s})\right)$, which for settings resulting in a large number of biclusters after the mining step (*k*≫*k*^′^) is approximately $\Theta \!\left (d\wp +{k \choose k/2}\bar {r}\bar {s}\right)$.

## Results

In this section, we present an extensive experimental evaluation showing that BicPAM is effective and computationally efficient. BicPAM was implemented in Java (JVM version 1.6.0-24). The following experiments were run in an Intel Core i3 1.80 GHz with 6 GB of RAM.

The results were collected and analyzed in four steps. Section “Comparison of biclustering approaches in synthetic data” compares the performance of BicPAM against state-of-the-art biclustering approaches. In Section “Performance analysis in synthetic data”, BicPAM’s behavior is extensively assessed in synthetic datasets with varying size, noise, sparsity and background distributions. The biological relevance of BicPAM’s results is analyzed in Section “Results in real data”. Finally, Section “Comparison of pattern-based biclustering approaches” goes further on comparing BicPAM and its pattern-based peers. Below, we describe the evaluation metrics and data settings used.

**Evaluation metrics.** Biclustering solutions have been assessed using multiple evaluation criteria. In the presence of hidden/planted biclusters, $\mathcal {H}=\{H_{1},..H_{g}\}$, clustering metrics^a^, match scores [2,58] and relative nonintersecting area (RNAI) [59,60] have been used. Match scores (MS) [58] assess the similarity of solutions based on the Jaccard index. $MS(\mathcal {B},\mathcal {H})$ defines the extent to what found biclusters match with hidden biclusters, while $MS(\mathcal {H},\mathcal {B})$ reflects how well hidden biclusters are recovered (1). RNIA [59] measures the overlap area between the hidden and found biclusters. To distinguish if several or few of the found biclusters cover a hidden bicluster, *clustering error* (CE) [60] is a critical extension. To take into account the number of biclusters in both sets, Hochreiter et al. [2] introduced a consensus score by computing similarities between all pairs of biclusters (2). We refer to this metric as FABIA Consensus (FC). Let *S*_1_ and *S*_2_ be, respectively, the larger and smaller set of biclusters from $\{\mathcal {B},\mathcal {H}\}$, and *MP* be the assigned pairs using the Munkres method based on overlapping areas [61], MC and FC are defined as:

(1) $$ \textbf{MS}(\mathcal{B},\mathcal{H})=\small\frac{1}{|\mathcal{B}|}\normalsize\Sigma_{(I_{1},J_{1})\in \mathcal{B}}{max}_{(I_{2},J_{2})\in \mathcal{H}}\footnotesize\frac{|I_{1}\cap I_{2}|}{|I_{1}\cup I_{2}|},  $$

(2) $$ {\footnotesize{\fontsize{9.3}{12}\begin{aligned} \textbf{FC}(\mathcal{B},\mathcal{H})=&\;\frac{1}{|\mathcal{S}_{1}|}\normalsize\Sigma_{((I_{1},J_{1})\in \mathcal{S}_{1},(I_{2},J_{2})\in \mathcal{S}_{2})\in MP}\\ &{\textstyle \times \frac{|I_{1}\cap I_{2}|\times |J_{1}\cap J_{2}|}{|I_{1}|\times |J_{1}| + |I_{2}|\times|J_{2}| - |I_{1}\cap I_{2}|\times |J_{1}\cap J_{2}|}.} \end{aligned}}}  $$

In the absence of hidden biclusters, merit functions can be used as long as they are not biased towards the merit criteria used within the approaches under comparison. Examples include the commonly used mean squared residue (MSR) [62] and its extension [16], or the Pearson’s correlation coefficent [59] sensitive to shifting-scaling properties. Finally, domain-specific evaluations can be used by computing statistical enrichment *p*-values in biological contexts [10,63].

**Data settings.** Gene expression data and two sets of synthetic datasets were used to evaluate BicPAM performance. The first set corresponds to the datasets generated by Hochreiter et al. [2]. These datasets simulate specific characteristics of gene expression data, such as heavy tail properties, using three settings: multiplicative models and additive models under signals according to *N*(±2,0.5^2^) and *N*(±4,0.5^2^) distributions [64]. Each setting has 100 datasets with 1000 genes, 100 conditions and 10 planted biclusters.

A second set of synthetic datasets with varying size and planted biclusters with varying degrees of expression was generated in the context of this work [65] (settings described in Table 1). We varied the size of the matrices up to 4.000 rows and 400 columns, maintaining the proportion between rows and columns commonly observed in gene expression data. The number and shape of the planted biclusters were also varied. The properties of the generated matrices were carefully chosen in order to follow properties from similar studies [10,13].

**Table 1:** **Properties of the generated set of synthetic datasets**

**Matrix size (** ***♯*** **rows ×** ***♯*** **cols)**	**100 × 30**	**500 × 60**	**1000 × 100**	**2000 × 200**	**4000 × 400**
Nr. of hidden biclusters	3	5	10	15	20
Nr. columns in biclusters	[5,7]	[6,8]	[6,10]	[6,14]	[6,20]
Nr. rows in biclusters	[10,20]	[15,30]	[20,40]	[40,70]	[60,100]
Area of biclusters	9.0%	2.6%	2.4%	2.1%	1.3%

The generated matrices were parameterized according to pre-specified number of items ($|\mathcal {L}|\in \{5,10,20\}$) and to an inputed bicluster type assumption (constant, additive, multiplicative and/or symmetric). The number of rows and columns for each bicluster followed a Uniform distribution over the ranges presented in Table 1. We allow for overlapping biclusters, which can difficult the recovery of the original planted biclusters. Finally, a noise factor was randomly added over the background values. This noise factor is up to ±15% of the range of values (e.g. *a*_*ij*_←*a*_*ij*_U(−1.5,1.5) when 10 items are available).

For each of these settings we instantiated 40 matrices: 20 matrices with background values following a Uniform distribution, $\mathrm {U}(1,|\mathcal {L}|)$, and 20 matrices with background values generated according to a Gaussian distribution, $\mathrm {N}\!\left (\frac {|\mathcal {L}|}{2},\frac {|\mathcal {L}|}{6}\right)$. The performance of BicPAM is an average across these 40 matrices.

### Comparison of biclustering approaches in synthetic data

We selected five state-of-the-art approaches able to discover biclusters under additive-multiplicative assumptions: FABIA with sparse prior option [2], Bexpa [66], ISA [67], Plaid [6] and OPSM [19]. Additionally, we considered CC [62], Samba [9], xMotifs [18], and three pattern-based biclustering approaches: BiModule [13], DeBi [10] and RAP [14]. Although the last six biclustering approaches use more simplistic homogeneity criteria, their inclusion is critical to study the biological significance of BicPAM’s solutions and to test BicPAM’s performance improvements when considering biclusters with constant models.

We used the following software to run these methods: R packages fabia [68] and biclust [69], BicAT [70], (Evo-)Bexpa [66] and Expander [71]. The specified number of biclusters for FABIA (with and without sparse equation), Bexpa, CC and ISA (number of starting points) was the number of hidden biclusters plus 10%: $|\mathcal {H}|\times 1.1$. Note that this required specification can be used to guide the search space exploration against other biclustering approaches and optimistically bias FABIA consensus (FC) levels. The default number of iterations for OPSM was varied from 10 to 200 iterations. The remaining methods were executed with default parameterizations. For this comparison, BicPAM was parameterized with closed patterns discovered using discretization methods with three distinct sets of items (|*Σ*|∈{3,5,7}), under a simple merging option (70% overlap) and filtering of biclusters based on an overlapping area over 30% against a larger bicluster. Additionally, two items were assigned to values near item-boundaries, leading to an increase in the size of transactions of 8-11%. The support threshold was incrementally decreased 10% and stopped when the discovered biclusters covered a minimum area of the input matrix (>5% ×|*X*|×|*Y*|).

**Figure 10:**
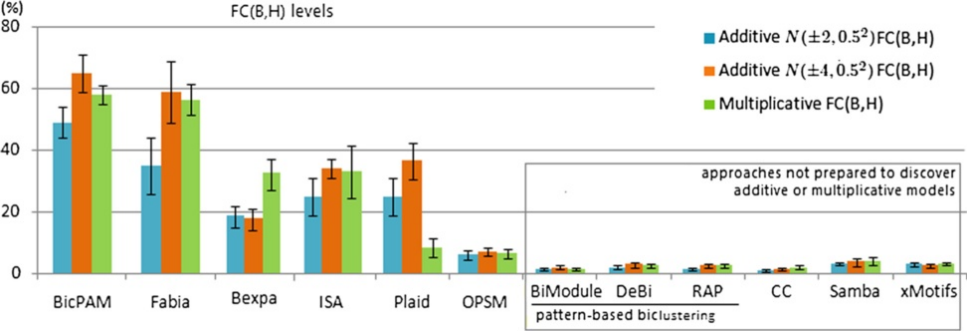
**FC levels across biclustering approaches using FABIA datasets.**

The ability of these approaches to retrieve the hidden biclusters using FABIA data settings is synthesized in Figure 10. FC$(\mathcal {B},\mathcal {H})$ was measured across the 100 matrices generated for each setting. BicPAM is the best performer for biclusters following additive models with different signal properties (Wilcoxon-test at 0.01%) and, together with FABIA, the best option for multiplicative models. The exhaustive nature of BicPAM searches and the ability to rely on multiple discretization levels without risk of introducing noise (by assignment multiple items for values near ranges-boundaries) support these observations. FABIA is a competitive non-exhaustive alternative, sensitive to the planted noise. Nevertheless, it requires prior knowledge regarding the number of biclusters. Since ISA is tunned to discover biclusters with gradual changes on values, its scoring schema to find modules with self-consistency is not well suited to discover biclusters modeled by additive signals. Plaid is able to locally identify additive factors. Understandably, the set of approaches not able to discover biclusters with scaling and shifting factors is considerably less effective. The FC levels of OPSM are strongly penalized since OPSM outputs a large number of biclusters with varying sizes (including biclusters with either small number of genes or conditions).

**Figure 11:**
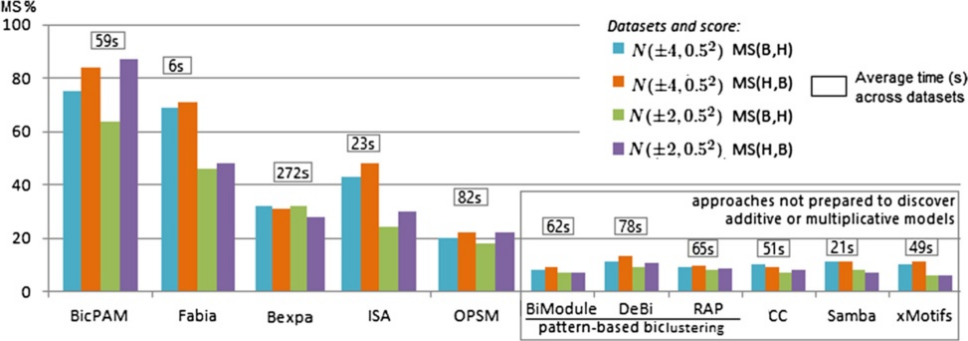
**Match scores across biclustering approaches using FABIA datasets.**

A comparison of match score levels across biclustering approaches when using the FABIA generated datasets is provided in Figure 11. Results confirm the superior performance of BicPAM both in terms of the MS$(\mathcal {B},\mathcal {H})$ score (correctness) and MS$(\mathcal {H},\mathcal {B})$ score (completeness). BicPAM is able to exhaustively mine the solution space and combine multi-level discretization thresholds. The average efficiency levels of BicPAM show its ability to perform exhaustive searches in useful time for computationally complex settings. FABIA is the most efficient approach.

**Figure 12:**
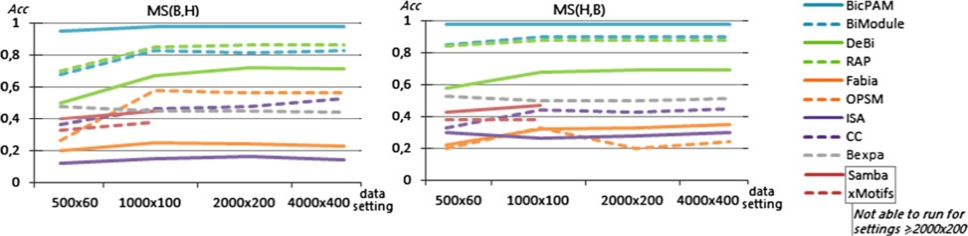
**Match scores of biclustering approaches using datasets with constant models.**

**Figure 13:**
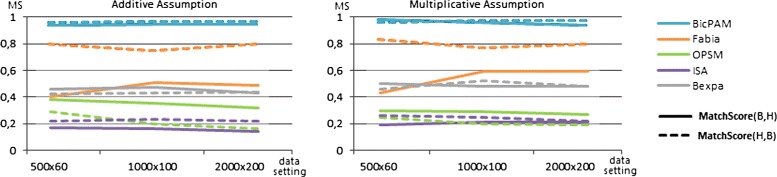
**Match scores of biclustering approaches using datasets with non-constant models.**

Figures 12 and 13 assess the ability of the analyzed biclustering approaches to discover planted biclusters with different coherency criteria (using an alphabet with 10 levels of expression) and varying the number of rows and columns (planted according to an Uniform distribution). Figure 12 shows that BicPAM’s performance (in the absence of extensions to discover non-constant biclusters) is superior against the three peer pattern-based methods. Figure 13 captures relevant changes in performance when considering additive and multiplicative coherencies. In order to promote the readability of these charts, we excluded the performance of the approaches not prepared to discover biclusters under these assumptions. Results confirm the superior performance of BicPAM in terms of MS$(\mathcal {B},\mathcal {H})$, that is, the majority of the discovered biclusters are well described by the hidden biclusters (correctness), and MS$(\mathcal {H},\mathcal {B})$, that is, the majority of the hidden biclusters can be mapped into a discovered bicluster (completeness). Although FABIA is the second choice for non-constant coherency, it is not prepared to deal with overlaps and it accommodates high levels of noise since it is not prepared to differentiate all of the 10 levels of expression, resulting in biclusters with a larger number of false positive genes.

**Figure 14:**
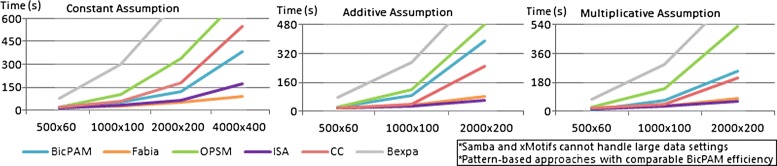
**Efficiency of biclustering approaches using the generated datasets.**

Finally, Figure 14 shows that, although all approaches are scalable for medium-sized matrices, efficiency deterioration is faster for OPSM, BicPAM and CC. The efficiency of peer pattern-based approaches is slightly worse than that of BicPAM as they do not seize the benefits of FP-growth searches.

### Performance analysis in synthetic data

In this section we study the efficiency limits of BicPAM. Then we assess the ability of BicPAM to discover different types of biclusters for data with varying regularities. Finally, we go further on understanding the impact of using different strategies related with the mining, mapping and closing steps.

#### Efficiency limits

**Figure 15:**
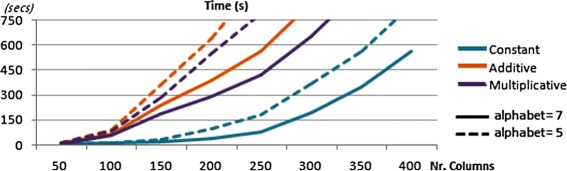
**Efficiency bounds of BicPAM for 10000 rows (magnitude of the human genome).**

To show the boundaries on BicPAM efficiency we considered matrices with 10.000 rows (magnitude of the human genome). The results are provided in Figure 15. We varied the number of conditions, the number of items ($|\mathcal {L}|\in \{5,7\}$) and the underlying coherency assumptions for this assessment. We consider the default merging procedure for the closing step. We planted 15 biclusters to occupy 2% of the area of the generated matrices and used Charm algorithm [45], an efficient pattern miner to deliver closed patterns (maximal biclusters). Generally, we observe that BicPAM is able to discover constant biclusters for matrices up to 10.000×350 and additive/multiplicative biclusters for matrices up to 10.000×200. Understandably the number of items has strong impact in efficiency as it defines the density of the correspondent itemset database and, therefore, the complexity of the mining step. Note, additionally, that the extensively studied scalability principles based on extensions over pattern mining methods – parallelization, distribution, streaming and error-bounding principles [12] – can be easily included in the mining step of BicPAM to guarantee its scalability over harder data settings.

#### Recovery of (non-)constant biclusters

**Figure 16:**
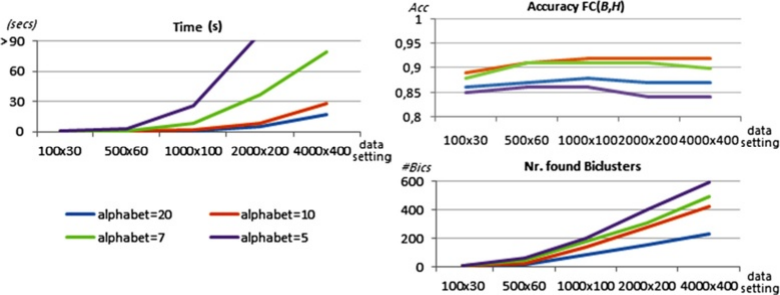
**Performance of BicPAM under a constant assumption.**

Although BicPAM relies on exhaustive searches, its performance highly depends on the ability to deal with noise, discretization errors and coherency assumptions. Figure 16 shows BicPAM’s performance with a parameterizable number of items for the datasets generated under a constant assumption. FC levels are attractive, although they are penalized by the exclusion of rows due to the planted noise, allowed overlapping among planted biclusters together with the fact that the number of discovered biclusters is usually higher than the number of planted biclusters.

**Figure 17:**
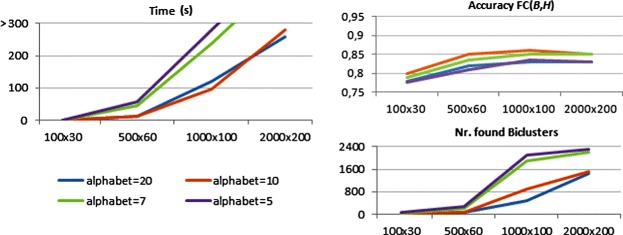
**Performance of BicPAM under an additive assumption.**

A smaller number of items turns the matrix denser, decreasing the efficiency bounds of BicPAM. Using a similar experimental setting, Figure 17 illustrates the performance of BicPAM for datasets with planted biclusters with an additive assumption. Although the observed FC scores are high, they are worse than for constant datasets due to the higher probability of background values to form a non-planted additive bicluster. Interestingly, although a na?ve search for additive biclusters would cost as much as |*Y*| times as the search for constant biclusters, the considered pruning fosters efficiency.

**Figure 18:**
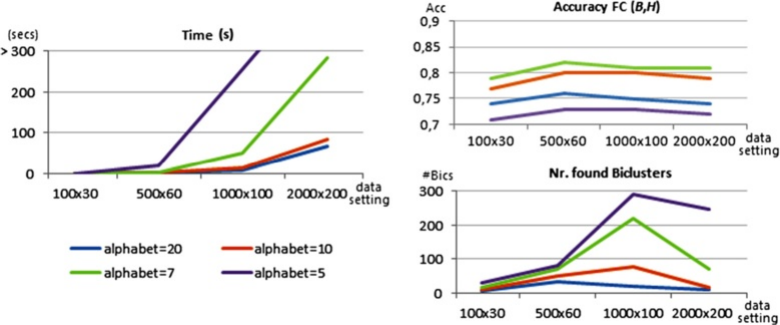
**Performance of BicPAM under a multiplicative assumption.**

Finally, Figure 18 illustrates BicPAM’s performance under a multiplicative assumption. Contrasting with the previous analysis, FC levels decrease for the larger matrices as the multiplicative factor is more prone to local mismatchings. This problem can, however, be corrected through closing options. Similarly to the search for additive biclusters, BicPAM seizes efficiency gains by pruning the search space. Additionally, the multiplicative assumption is structurally more efficient than its additive peer since the number of spurious biclusters is considerably low due to the broader range of items observed within each iteration, which leads to sparser matrices.

**Figure 19:**
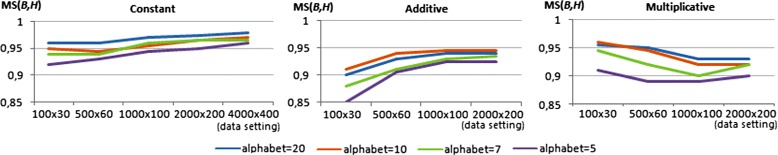
**Match score levels of BicPAM under constant, additive and multiplicative assumptions.**

To complement previous analysis, Figure 19 provides BicPAM’s MS$(\mathcal {B},\mathcal {H})$ levels for different levels of expression. The observed MS levels are higher than FC levels due to the absence of penalizations of outputting more biclusters than the number of planted biclusters. In particular, MS levels for medium- to-large datasets are, respectively, above 95%, 91% and 87% for constant, additive and multiplicative.

A detailed look of BicPAM’s performance, when considering 7 items and default noise handling, merging and filtering options, is provided in Table 2. The results are organized according to bicluster type, matrix size (and structure of planted biclusters) and underlying distribution of background values. The slightly worse performance when the input values are generated by a Gaussian distribution is not related with the increased probability of background values to form non-planted biclusters (since values are properly discretized), but with the increased difficulty of modeling the planted biclusters with Uniform values. We found MS$(\mathcal {B},\mathcal {H})$ to be lower than MS$(\mathcal {H},\mathcal {B})$ since the exhaustive nature of BicPAM leads to at least one found bicluster with a direct correspondence to each hidden bicluster.

**Table 2:** **FC and MS levels of BicPAM in different settings (mean and variance from 20 datasets)**

		**100 × 30**		**500 × 60**		**1000 × 100**		**2000 × 200**	
**Metric**	**Coherency**	**Normal**	**Uniform**	**Normal**	**Uniform**	**Normal**	**Uniform**	**Normal**	**Uniform**
FC	Constant	0.862 ±0.017	0.930 ±0.014	0.884 ±0.018	0.956 ±0.007	0.909 ±0.017	0.949 ±0.006	0.907 ±0.014	0.948 ±0.011
	Additive	0.782 ±0.021	0.831 ±0.008	0.834 ±0.014	0.888 ±0.007	0.845 ±0.018	0.897 ±0.007	0.827 ±0.015	0.887 ±0.006
	Multiplicative	0.762 ±0.028	0.794 ±0.013	0.790 ±0.019	0.825 ±0.014	0.785 ±0.020	0.840 ±0.011	0.767 ±0.020	0.819 ±0.015
MS$\left (\mathcal {B},\mathcal {H}\right)$	Constant	0.923 ±0.018	0.974 ±0.007	0.931 ±0.012	0.968 ±0.005	0.935 ±0.010	0.984 ±0.005	0.944 ±0.011	0.987 ±0.008
	Additive	0.895 ±0.017	0.945 ±0.006	0.925 ±0.012	0.963 ±0.003	0.913 ±0.008	0.981 ±0.007	0.917 ±0.011	0.974 ±0.006
	Multiplicative	0.902 ±0.019	0.958 ±0.014	0.906 ±0.015	0.953 ±0.009	0.910 ±0.015	0.941 ±0.008	0.886 ±0.019	0.948 ±0.010
$MS\left (\mathcal {H},\mathcal {B}\right)$	Constant	0.956 ±0.013	0.984 ±0.006	0.960 ±0.007	0.981 ±0.004	0.961 ±0.004	0.996 ±0.002	0.957 ±0.009	0.993 ±0.002
	Additive	0.955 ±0.012	0.997 ±0.001	0.959 ±0.006	0.997 ±0.002	0.955 ±0.004	0.995 ±0.002	0.957 ±0.007	0.995 ±0.003
	Multiplicative	0.937 ±0.015	0.966 ±0.008	0.924 ±0.012	0.968 ±0.008	0.923 ±0.010	0.963 ±0.009	0.927 ±0.013	0.974 ±0.007

#### Mining options

**Figure 20:**
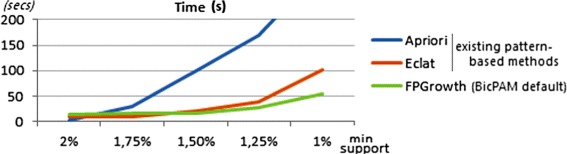
**Comparison of pattern mining algorithms for the 1000×100 setting.**

Figure 20 illustrates the impact of the algorithmic choice in the efficiency of BicPAM. The three main paradigms for frequent itemset mining (Apriori, FPGrowth, and vertical-based Eclat) were tested based on implementations from *SPMF* [72] software. These methods were extended in order to be able to deliver the transaction set supporting each frequent itemset. For this assessment we used a discretization step with 10 items and constant planted biclusters based on all frequent patterns. The results were collected for the 1000×100 generated dataset setting. FPGrowth and Eclat are the most competitive choices when dealing with very small support thresholds. In particular, FPGrowth is the best performer for the setting used for supports near and below 1%. Finally, Apriori is the best option for medium-to-large support levels.

**Figure 21:**
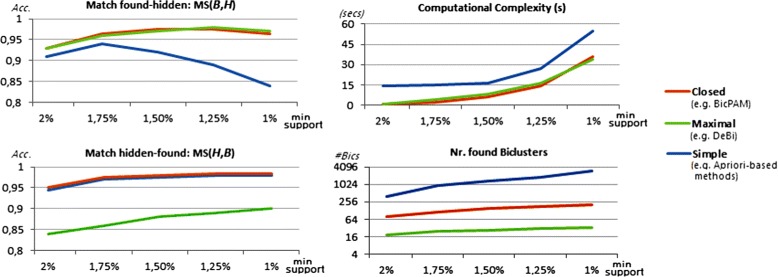
**Impact of choosing alternative pattern representations over the 1000 × 100 data setting.**

The impact of choosing alternative pattern representations (simple, closed, maximal) in efficiency and MS levels is presented in Figure 21. For this assessment we used three distinct methods: FPGrowth [42] to output simple patterns, Charm [45] to output closed patterns (maximal biclusters) and CharmMFI [45] to output maximal patterns. Similarly, we considered the 1000×100 setting and 10 items.

Three main observations can be retrieved from this analysis. First, the use of maximal patterns for biclustering should be avoided as it gives preference to biclusters with a large number of columns and discards biclusters with a subset of these columns (even when they have a larger number of rows). Understandably, this penalizes the $MS(\mathcal {H},\mathcal {B})$ levels. $MS(\mathcal {B},\mathcal {H})$ scores are not so affected as each maximal bicluster is covered by a planted bicluster. Second, the use of simple patterns for biclustering can degrade the $MS(\mathcal {B},\mathcal {H})$ in comparison with closed patterns. This score penalizes the discovery of biclusters contained in larger planted biclusters, even when the discovered biclusters have a heightened homogeneity. Third, the search for closed and maximal patterns is slightly more efficient than the search for simple patterns as a result of additional pruning procedures. These observations support the use of closed patterns. Furthermore, they correspond to maximal biclusters, which are in general the aim of effective biclustering algorithms [1,13,73].

#### Mapping options

**Figure 22:**
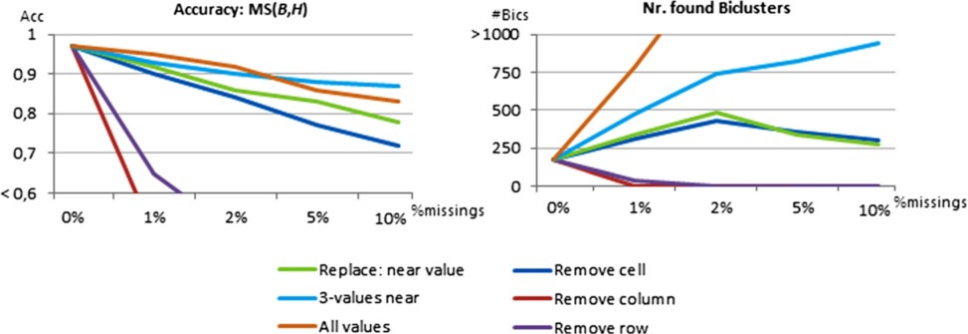
**Comparing the handling of missings for data with varying levels of noise.**

In order to assess the impact of the proposed mapping strategies to handle *missing values* (Figure 6), we randomly removed a varying number of elements from the generated matrices for the 1000×100 setting. Figure 22 illustrates how the performance of BicPAM (using Charm and 10-item discretization) varies with a percentage of missings ranging from 0 to 10% (that is, from 0 to 10.000 elements). Note that 10% is already considered a very critical number of missings that may compromise the ability to retrieve the true biclusters. We observe that this problem can be mitigated recurring to the proposed BicPAM missing handlers.

When analyzing the results in Figure 22, three observations can be retrieved. First, $MS(\mathcal {B},\mathcal {H})$ under the baseline strategy (remove the missings) significantly decreases from 97% to near 70% when the percentage of missings reaches 10%. Although this solution is easily implemented in BicPAM (removing an element from respective transactions), the majority of existing biclustering algorithms only allow for removals on the columns or the rows where a missing occurs (impracticable even in the presence of a few missings as illustrated). Second, the ability to retrieve the planted biclusters increases when considering the nearest 2-3 values against the strategies that consider the closest value only or all the possible values (relaxed strategy). This is justified by two factors: *1)* when estimating more than one value for a missing, there is an increased chance to recover the original value and, therefore, of not damaging a planted bicluster; *2)* when considering all the possible values for a missing, there is an increased amount of noise that is added and can lead to the emergence of false biclusters. Third, although inserting multiple values to replace a missing is an attractive option in terms of accuracy, its efficiency is penalized as the itemized matrix becomes denser (consistent with the number of discovered biclusters). Still, when considering only the closest 2-3 values, scalability is maintained for levels of noise up to 10%.

#### Closing options

We planted additional levels of *noise* to evaluate the closing options. This was performed by changing the values of specific elements by a randomly distant value (distance >25% of the domain range). The percentage of noisy elements was varied from 0 to 10%. We used the 1000×100 setting, Charm and a total of 10 items.

**Figure 23:**
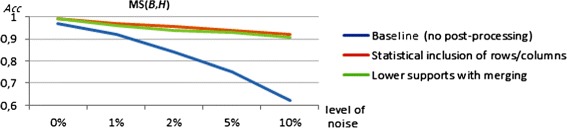
**Impact of extending biclusters for data with varying levels of noise.**

Figure 23 describes the impact of alternative strategies to *extend* biclusters. When no noise is planted, merging-based strategies are able to achieve slightly higher matching scores since they can cover elements originally missed due to discretization errors or by the allowed overlapping among planted biclusters. When increasing the planted noise, the presence of extension options is critical to maintain interesting accuracy levels. Both the inclusion of new rows and columns (recurring to statistical tests or by lowering the support of pattern miners) and the merging of the resulting biclusters are able to maintain match scores above 90% (20 percentage points higher than the baseline option).

**Figure 24:**
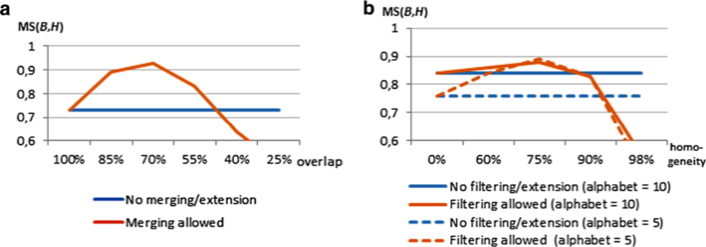
**Impact of merging and filtering (reduction) for the 1000×100 setting.** **(a)** Merging for varying overlapping degrees (5% of planted noise). **(b)** Filtering for varying homogeneity degrees (2% of planted noise).

Figure 24(a) illustrates the impact of *merging* biclusters with large overlapping areas assuming a level of planted noise of 5%. The baseline case corresponds to an overlapping area of 100%. When relaxing the overlapping criteria, $MS(\mathcal {B},\mathcal {H})$ (and also $MS(\mathcal {H},\mathcal {B})$) increases, as the merging step allows for the recovery of missing rows and columns. However, this improvement in behavior is only observable until a certain overlapping threshold (near 70% for this experimental setting). Match scoring decreases below this threshold. A correct identification of the optimum threshold can lead to significant gains (near 15 percentage points for this experimental setting).

Finally, the use of *filtering* strategies can also lead to an enhanced ability to recover the planted biclusters. Although the filtering of biclusters with weak homogeneity impacts accuracy, this analysis targets the removal of rows and columns (on each bicluster) that do not satisfy a specific homogeneity threshold. Figure 24(b) illustrates the impact of removing potentially false rows and columns assuming a level of planted noise of 2%. The impact is only significant when considering a low-to-medium number of items, since for these cases filtering is able to correct the errors related with the large ranges of values per item that lead to false biclusters. Similarly to the merging option, an increase in the matching score is observed when compared to the baseline case (an homogeneity degree of 0%) up to 75%, given by 1 −*MSR* [62]. From this upper threshold the match scores decrease since the homogeneity criteria becomes too restrictive.

### Results in real data

To assess the performance of BicPAM in real data, we compared the biological significance of BicPAM’s solutions against state-of-the-art biclustering solutions using three distinct gene expression datasets [74,75]: *1)dlblc* dataset (660 genes, 180 conditions) to study responses to chemotherapy [76], *2)hughes* dataset (6300 genes, 300 conditions) to characterize nucleosome occupancy [77], and *3)gasch* dataset (6152 genes, 176 conditions) to measure Yeast responses to environmental stimuli [78]. For the *gasch* dataset, we considered the multiple time points per condition and averaged the replicates of the steady state. The missing values were not removed since BicPAM can cope with them. For the state-of-the-art biclustering approaches, we maintained the parameterizations used in the previous section. In particular, pattern-based approaches were parameterized with multiple levels of expression ($|\mathcal {L}|\in \{4..7\}$). BicPAM output include constant, additive, multiplicative and symmetric biclusters, discovered under different closing options. The selected closing options were: merging (70% overlap); relaxed merging (55% overlap) with filtering of rows; and tight merging (90% overlap) with extensions on rows that appear in another bicluster sharing a minimum 50% of the conditions. In what follows, we analyze the results obtained focusing the three following points: 1) functional enrichment, 2) transcriptional regulation, and 3) coherence.

#### Functional enrichment

The biological relevance of the biclusters from the different biclustering solutions was obtained using the Gene Ontology (GO) annotations computed by GoToolBox [79]. To discover the enriched GO terms, we computed the p-values obtained using the hypergeometric distribution to access the over-representation of a specific term. In order to consider a bicluster to be significant, we require its genes to show enrichment in one or more of the “biological process” ontology terms by having a (Bonferroni corrected) p-value below 0.05.

Table 3 provides a compact view on the biological significance of the compared approaches. BicPAM is able to discover the largest number of (non-similar) biclusters with significantly enriched terms for each dataset. The analysis of these terms against the significant terms found in other biclustering solutions shows the completeness of BicPAM’s solutions (as they cover the majority of the gathered biological functions per dataset), together with the exclusivity and relevance of BicPAM solutions (as they model biclusters with significantly enriched GO-terms that are not discovered by the remaining approaches). Although peer pattern-based solutions also find a large number of biclusters with significantly enriched terms, these terms have lower significance. This is due to the fact that these approaches do not provide noise-correction procedures to minimize the item-boundaries problem and cannot discover non-constant biclusters. Additionally, the remaining biclustering solutions provide incomplete sets of GO-terms since their algorithms are not able to deliver flexible biclustering structures with multiple coherencies. Moreover, some of these approaches are neither able to discover biclusters with multiple levels of expression (or homogeneity levels) nor postprocess the raw biclustering solutions. Still, some of the compared approaches were able to deliver a few small biclusters whose terms are more significant than those found with BicPAM. Subsequent analyzes (Tables 4, 5 and 6) provide further empirical evidence for the relevance, completeness and exclusivity of BicPAM solutions.

**Table 3:** **Comparing the biological relevance and novelty of different biclustering solutions**

**Dataset**	**Approach**	***♯*** **Bics**	**Avg.** ***♯*** **Genes ×** ***♯*** **Conds**	***♯*** **Bics sig.** **enriched**	**Coverage and exclusivity of enriched GO terms**
*dlblc*	BicPAM	56	83 ×7	43 (77%)	Highest number of exclusively enriched terms (partial list in Table 4).
*(human*	BiModule	322	62 ×4	79 (25%)	Absence of closing options leads to redundant and less significant terms.
*genome)*	DeBi	31	73 ×6	21 (68%)	Loss of relevant terms due to the inability to discover all maximal biclusters.
	CC	10	41 ×33	5 (50%)	Exclusive bicluster related with circulatory & cardiovascular system development.
	ISA	72	23 ×8	8 (11%)	Exclusive bicluster for extracellular structure organization and heparin binding.
	Plaid	3	12 ×49	1 (33%)	Majority of genes modeled in a single background bicluster with general terms.
	Fabia	10	79 ×35	6 (60%)	Small bicluster with superior enrichment of antigen binding functions.
	Bexpa	10	16 ×87	2 (20%)	Small sets of genes supported by large number of conditions.
	Samba	100	17 ×6	18 (18%)	Dedicated terms for antigen processing, peptide cross-linking and disassembly.
	OPSM	12	128 ×5	5 (42%)	High variance of *♯*genes and *♯*conditions; some of the biclusters with low *♯*genes (coherency across high *♯*conditions) have exclusive significantly enriched terms.
*hughes*	BicPAM	47	360 ×7	38 (81%)	Exclusive enriched terms due to flexible coherency and post-processing criteria.
*(yeast*	BiModule	219	285 ×4	43 (20%)	Terms with lower sig. than terms from noise-tolerant BicPAM solutions.
*genome)*	DeBi	28	317 ×7	21 (75%)	Terms observed across very small sets of conditions (≤5) are not enriched.
	CC	10	228 ×58	6 (60%)	GO terms covered by BicPAM constant biclusters.
	ISA	8	120 ×4	5 (63%)	Small biclusters with exclusive significance GO terms: spindle pole and karyogamy.
	Plaid	8	78 ×39	3 (38%)	One bicluster with higher significance for fungal-type cell wall assembly.
	Fabia	10	210 ×49	5 (50%)	Higher significance observed for actin cortical patch and oxidoreductase GO-terms.
	Bexpa	72	42 ×49	1 (10%)	Low number of enriched terms (probably due to the low *♯*genes per bicluster).
	Samba	120	18 ×9	11 (9%)	Enriched terms covered by pattern-based biclustering solutions.
	OPSM	6	531 ×4	3 (50%)	Exclusive bicluster for the negative regulation of metabolic processes.
*gasch*	BicPAM	149	411 ×8	123 (83%)	Large diversity of highly significant GO-terms (partial list in Table 4).
*(yeast*	BiModule	653	287 ×4	159 (24%)	Large but incomplete set of GO-terms as it excludes non-constant biclusters.
*genome)*	DeBi	82	310 ×6	61 (74%)	Significance of terms slightly differ than BicPAM due to the handling of noise.
	CC	10	203 ×79	7 (70%)	Enriched terms appear in BicPAM solutions with higher significance.
	ISA	23	292 ×22	18 (78%)	Enriched terms covered by pattern-based biclustering solutions.
	Plaid	6	48 ×12	3 (50%)	Biclusters (apart from background layer) with lower enrichments than peers.
	Fabia	10	310 ×41	8 (80%)	Bicluster with higher sig. for specific proteasome complexes.
	Bexpa	10	63 ×29	3 (33%)	The few biclusters with deviation in size (higher *♯*genes) are significant.
	OPSM	16	212 ×8	11 (69%)	One bicluster with higher significance for pre-ribosome functions.

**Table 4:** **Summary on the biological relevance of BicPAM’s biclusters**

**Dataset**	**Closing option**	***♯*** **Bics**	**Avg. Area**	***♯*** **Filtered bics**	***♯*** **Highly sig. bics**	***♯*** **Sig. bics**
	merging	4803	81 ×7	28	22	5
*dlblc*	relaxed *merging* + reductions	980	83 ×9	24	19	3
	tight *merging* + extensions	7652	79 ×6	27	25	2
	merge	6311	432 ×6	36	19	12
*hughes*	relaxed *merging* + reductions	1259	492 ×7	22	12	8
	tight *merging* + extensions	9210	398 ×5	39	22	11
	merge	27031	392 ×8	89	66	12
*gasch*	relaxed *merging* + reductions	2177	486 ×11	67	49	11
	tight *merging* + extensions	52123	367 ×7	92	79	9

**Table 5:** **Terms highly enriched in BicPAM’s biclusters**

**Dataset**	**ID**	**Terms**	**Bicluster with best** ***p*** **-value**	***♯*** **Genes**
*dlblc*	Dl1	translational elongation; cytosolic part; translational initiation	4.49E-5	81
	Dl2	Golgi apparatus; MHC protein complex	5.40E-5	83
	Dl3	defense response; receptor activity; single organism signaling; vacuole; cell communication	4.91-5	162
	Dl4	immune response; response to interferon-gamma	1.06E-4	58
	Dl5	immune system process	1.27E-4	52
	Dl6	response to interferon-gamma; cellular response to chemical stimulus; response to cytokine stimulus	0.001	60
	Dl7	membrane-enclosed lumen; cell division; cell cycle process	2.92E-12	81
	Dl8	small molecule binding; catalytic activity; cell cycle process	6.14E-8	108
*hughes*	H1	mitochondrion organization; organellar ribosome; mitochondrial matrix; mitochondrial translation	2.70E-39	416
	H2	cell periphery; cell wall constituent; oxidoreductase activity; cell wall organization; sexual sporulation	1.73E-4	370
	H3	ribonucleoprotein complex biogenesis; nucleus	3.61E-30	426
	H4	cellular amino acid metabolic/biosynthetic process; carboxylic acid metabolic/biosynthetic process	1.3E-25	581
	H5	organonitrogen compound metabolic process; sulfur compound metabolic process	1.62E-4	504
	H6	macromolecular complex; intracell. non-membrane-bounded organelle; membrane-enclosed lumen	4.80E-14	512
*gasch*	G1	nitrogen compound metabolic proc.; carboxylic/organic amino acid processes; structural cytoskeleton	1.84E-16	434
	G2	cellular carbohydrate metabolic process; cytoplasm	2.01E-7	265
	G3	generation of precursor metabolites and energy; tricarboxylic acid cycle	1.16E-14	954
	G4	endomembrane system; retrotransposon nucleocapsid; pore; viral procapsid maturation	4.34E-6	102
	G5	nucleolus; ncRNA metabolic process	1.03E-61	611
	G6	intracell. non-membrane-bounded organelle; structural molecule activity	5.33E-76	293
	G7	cytosolic part; ribosomal subunit	1.61E-88	460
	G8	membrane-enclosed lumen; nuclear lumen; intracell. organelle lumen	1.17E-47	263
	G9	mitochondrion organization; mitochondrial part; cytoplasmic part; protein complex biogenesis	2.06E-26	592
	G10	cellular response to oxidative stress; generation of precursor metabolites and energy	2.37E-4	296
	G11	binding; nuclear part; preribosome	2.87E-11	508
	G12	cellular process involved in reproduction	0.001	435
	G13	macromolecular complex; cell part; structural molecule activity	6.05E-29	1442
	G14	vacuolar transport; chromosome	5.09E-7	606
	G15	regulation of cellular (macromolecule) biosynthetic process; protein modification process	2.28E-13	1019
	G16	organic substance catabolic process; carbohydrate metabolic process; cytoplasm	1.02E-15	648
	G17	ribonucleoprotein complex biogenesis (general)	1.08E-94	784

**Table 6:** **Illustrative set of biclusters with different properties and heightened biological relevance (** ***p*** **-values after Bonferroni correction)**

**Dataset**	**ID**	**Pattern**	**Items**	**Closing options**		
	B1	FAABFFF	A-F	Merging with tight overlapping		
*dlblc*	B2	AAABCA	A-C	Extensions allowed (with tight merging)		
	B3	AAA/../EEE	A-E	Reducing with high homogeneity		
	B4	EEECEE	A-E	Merging allowed		
*hughes*	B5	CCDCBCBCC	A-E	Merging with relaxed overlapping		
	B6	AAAAA/../G..G	A-G	Merging with tight overlapping		
*gasch*	B7	AAAGGGA	A-G	Merging with tight overlapping		
	B8	AAABACCCAA	A-E	Merging allowed		
**ID**	**Type**	***♯*** **Genes**	***♯*** **Conds**	***♯*** ***p*** **−values <0.01**	***♯*** ***p*** **−values [0.01,0.05]**	**Best p-value**
B1	constant	83	7	41	21	1.97E-10
B2	constant	153	8	9	1	2.27E-12
B3	multiplicative	119	5	5	18	4.12E-8
B4	constant	581	6	12	7	1.31E-25
B5	constant	654	10	16	4	1.31E-17
B6	additive	476	6	12	10	1.92E-6
B7	multiplicative	483	7	57	10	1.24E-81
B8	additive	521	10	17	5	4.57E-12

Table 4 shows the number of biologically significant biclusters found by BicPAM when using closing strategies. In this analysis, a bicluster is considered to be highly significant if it has at least one enriched term with a corrected p-value below 0.01. To complement this analysis, Table 5 lists some of the most significant biological processes associated with these enriched terms for each data setting [80].

Table 6 shows an illustrative set of the found pattern-based biclusters with statistical relevance. Such biclusters could hardly be discovered by peer biclustering methods, since many of them include conditions with multiple degrees of expression (B1, B2 and B5) and non-constant profiles (B8). All of these biclusters have heightened biological significance as observed by the number of highly enriched terms after Bonferroni correction. Interestingly, we also observe that different closing options lead to biclusters with different shapes, even when the number of items is the same (B4 and B5).

Although a detailed biological analysis is out of the scope of this paper, we provide a brief analysis for one bicluster per dataset. The bicluster identified in Tables 6 and 7 as *B1*, with 83 human genes with coherent expression across 7 samples, was discovered in *dlblc* using 6 levels of expression (under a Gaussian discretization). These genes showed very low expression (A) on 2 samples, low expression (B) on 1 sample and very high expression (F) on 4 samples. Over 40 GO terms were highly significant, with the top set of terms being related with immune defense responses (e.g. immune system process, regulation of immune system process) and signaling functions associated to immunomodulating agents, such as cytokine. Significant terms related with Golgi and with the formation of membrane-bound compartments imply their critical roles during the induction of innate immune responses after chemotherapy [81]. Similar biclusters are not discovered when the number of expression levels is decreased or when noise relaxations are not included, thus motivating the need for BicPAM. The illustrative biclusters, found in *hughes* and *gasch* datasets, concern genes from Saccharomyces cerevisiae analyzed in the context of studying nucleosome occupancy and responses to different stress conditions, respectively. The enriched terms of bicluster B4 are associated with the formation of carboxylic acid and organonitrogen compounds, with optimum enrichment levels found in the presence of moderate noise-tolerance. Bicluster B7 captures genes with coherent expression across multiple time points from three different heat shocks (shocks from 17, 21 and 25°C). The analysis of GO terms shows functions related with the ribonucleoprotein complex (*p*-value 1.24E-81), associated with the reassembly and protection of small particles during heat stress responses [82]. Interestingly, other biclusters found in *gasch* are able to capture coherent levels of expression across different stimuli. An example is bicluster B8 that integrates conditions related with nitrogen depletion, heat stress and diauxic shift. B8 has 521 genes, coherent additive levels of expression across 10 conditions, and over 10 highly significant enriched terms.

**Table 7:** **Enriched GO terms of three illustrative BicPAM biclusters**

**ID**	**Dataset**	**Top 4 GO Terms (** ***p*** **-value)**
B1	*dlblc*	Immune response (2.32E-10); immune system process, defense response (<1E-6);
		cytokine-mediated signaling pathway (1.33E-7); Golgi apparatus (1.19E-7).
B4	*hughes*	Carboxylic acid biosynthetic process (1.3E-25) and metabolic process (6.12E-16);
		organonitrogen compound biosynthetic process (2.23E-18) and metabolic process (2.71E-13).
B7	*gasch*	Ribonucleoprotein biogenesis and assembly (1.24E-81); cytosolic part (1.22E-57);
		intracell. non-membrane-bounded organelle (1.31E-65); ncRNA metabolic process (1.82E-52).

#### Transcriptional regulation

To complement the results on functional enrichment, we analyzed the highly enriched transcription factors (TFs) using the TFCONES database [83] (human genome) and Yeastract database [84] (yeast genome) using a corrected hyper-geometric statistical test.

Consider the illustrative biclusters provided in Table 7. Some of the enriched transcription factors regulating the genes in bicluster *B*1 (associated with immune system responses in the human genome) include: HCLS1 gene that plays a key role in regulating clonal expansion and deletion in lymphoid cells [85], IRF1 protein that acts as a tumor suppressor and plays a role not only in antagonism of tumor cell growth but also in stimulating an immune response against tumor cells [85], and TRIM22 antiviral protein involved in cell innate immunity [83]. Other highly enriched TFs that regulate proliferation and transformation (tumor supressors) are ANP32A and RUNX3 [85]. The TFs regulating the genes in bicluster *B*4 have *p*-values below 1E-15 after correction, each regulating from 50% to 95% of the genes in bicluster. They are associated with regulatory functions consistent with the enriched terms. Some of these TFs include histidine biosyntehsis (Bas1p), amino acid biosynthesis (Gcn4p), cyclic AMP receptor protein regulation (Sok2p) and other TFs related with the regulation of carboxylic acid and organonitrogen compounds [86]. Consider now bicluster *B*7 from *gasch*. Some of the enriched TFs include Sfp1p, Mga2p, Ace2p, Tup1p, Spt10p and Swi5p (*p*-values below 1E-15), each regulating 55%-97% of B7’s genes. These factors are known to be involved in stress responses as they regulate cooling and oxygen levels (Mga2p), repair cellular damage (Sfp1p and Spt10p), remodel chromatin (Tup1p) and regulate cell wall protection (Swi5p and Ace2p) [86–88]. Finally, consider bicluster *B*8, whose genes coherently regulate heat, nitrogen depletion and diauxic shifts. Sfp1p, Bas1p, Ste12p and Tec1p were the most significant TFs in this bicluster (*p*-values <1E-7). Sfp1p controls expression of ribosome biogenesis genes in response to stress and DNA-damage response [86]. Bas1p regulates gene expression for biosynthesis pathways such as pathways related with histidine metabolism, which responds to environmental stimuli (e.g. nitrogen) affecting pH calibration [86]. Finally, Ste12p and Tec1p act together to regulate genes related with invasive growth, whose production is expected under such stress conditions [86].

An extended analysis of the TFs associated with BicPAM’s biclusters for the human and yeast genome is provided in Table 8. In this analysis we retrieved the TFs that are more *representative* – high coverage of the genes in the biclusters – and *significant* – high functional enrichment (*p*-value <1E-3) – for each one of the twenty five distinct biclusters disclosed in Table 5 associated with the *dlblc* and *gasch* dataset. In line with the goal of the these experiments [76,78], we observe that the identified TFs are either directly or indirectly related with the responses to chemotherapy (human) [83,85] and stress conditions (yeast) [84,86]. This analysis thus further supports the domain-relevance and adequacy of BicPAM.

**Table 8:** **Analysis of TFs of the putative regulatory modules given by the BicPAM’s biclusters provided in Table** **5** **for the human genome (** ***dlblc*** **dataset) and the yeast genome (** ***gasch*** **dataset)**

***Dataset***	***Bic.ID*** ** (Table ** **5** **)**	***Highly enriched TFs***
dlblc	Dl1	BCL11A, LZTS1, GTF2I, HCLS1, HDAC1, MBD4, MEF2B, NCOA3, STAT6
	Dl2	ANP32A, HCLS1, IRF1, MNDA, NCOA1, RUNX3, STAT1, TRIM22, TRIP10
	Dl3	BCL3, TRIM22, ANP32A, ARID5B, CEBPB, CREG1, IRF1, PFDN5, STAT1
	Dl4	ANP32A, IRF1, NCOA1, STAT1, TRIM22
	Dl5	CREG1, IRF1, TRIM22, ANP32A, STAT1
	Dl6	ANP32A, IRF1, NCOA1, STAT1, TRIM22
	Dl7	BCL6, BCL6B, HIf1A, ILF2, POU2AF1, SERTAD1, TCF3
	Dl8	DR1, DRAP1, HIf1A, ILF2, NCOA3, SERTAD1, TMF1, ZNFN1A1
gasch	G1	Gcn4p, Sfp1p, Ace2p, Tec1p, Ste12p, Ash1p
	G2	Sfp1p, Msn2p, Bas1p, Tec1p, Sok2p, Abf1p, Ash1p, Cst6p
	G3	Sfp1p, Tec1p, Ste12p, Msn2p, Bas1p, Sok2p, Msn4p, Gcn4p
	G4	Snf6p, Tec1p, Ste12p, Rap1p, Sin4p, Abf1p, Snf2p, Ash1p
	G5	Sfp1p, Ace2p, Cst6p, Tup1p, Msn2p, Spt10p, Spt20p
	G6	Hsf1p, Spt23p, Mga2p, Sfp1p, Spt10p, Msn2p, Gcr1p, Gcn4p
	G7	Sfp1p, Swi5p, Tup1p, Spt10p, Spt20p, Gcr1p, Sin3p, Mga2p
	G8	Sfp1p, Swi5p, Cst6p, Tup1p, Spt20p, Ash1p, Spt10p
	G9	Yap1p, Ace2p, Sfp1p, Msn2p, Ash1p, Msn4p, Abf1p
	G10	Sfp1p, Msn2p, Msn4p, Cst6p, Abf1p, Sok2p, Bas1p
	G11	Snf6p, Tup1p, Snf2p, Cst6p, Sin4p, Rap1p, Swi3p, Hap2p
	G12	Yap1p, Tec1p, Msn2p, Msn4p, Ste12p, Sok2p
	G13	Snf6p, Tup1p, Abf1p, Snf2p, Cst6p, Sin4p
	G14	Sfp1p, Tec1p, Ste12p, Bas1p, Sok2p, Yrm1p
	G15	Ace2p, Sfp1p, Tec1p, Ste12p, Ash1p, Bas1p, Gcn4p, Sok2p
	G16	Cin5p, Gcn4p, Msn4p, Sfp1p, Msn2p, Tec1p, Ste12p, Sok2p
	G17	Sfp1p, Ace2p, Cst6p, Snf6p, Rap1p, Tup1p, Spt10p, Swi5p

Consider the enriched TFs provided in Table 8 for a sample set with 8 distinct biclusters found by BicPAM in the *dlblc* dataset. Different groups of TFs were identified, each associated with a specific chemotherapy outcome. Some of the TFs acting as putative tumor suppressors include: ANP32A, LZTS1 (protein-coding silenced in rapidly metastasizing and metastatic tumor cells), RUNX3 (protein that binds to the core site of leukemia virus, also frequently silenced in cancer), HCLS1 (antigen receptor signaling deletion in lymphoid cells), IRF1 (protein that stimulates immune responses and regulates tumor cell differentiation), HIf1A (gene responsible for tumor angiogenesis and pathophysiology of ischemic disease), HDAC1 (complex interacting with retinoblastoma tumor-suppressor proteins), TCF3 (protein regulating lymphopoiesis as its deletion is associated with lymphoblastic and acute leukima malignancies) [83,85]. Other TFs dedicated to regulate cell proliferation include the STAT families, CREG1, MEF2B, ARID5B, and BCL3 [85]. Understandably, we also observed the B-cell lymphoma protein (BCL6 and its paralog coding gene BCL6B) and other leukemia-related disease genes involved in lymphoma pathogenes, such as BCL11A [83]. Complementarily, immune responses are associated with TRIM22 antiviral proteins, CEBPB, NFATC2 complex, and GTF2I for activating immunoglobulin heavy-chain transcription upon B-lymphocyte activation [85].

Finally, consider the enriched TFs provided in Table 8 for a sample set with 17 distinct biclusters found by BicPAM in the *gasch* dataset. Since a large number of enriched TFs was identified, Table 8 only provides an illustrative set containing TFs regulating over 50% of the genes associated with each bicluster. Although the enriched TFs regulate very distinct processes (see Table 5), most TFs are activated in stress conditions, namely: Yap1p, Cin5p and Hap2p during oxidative stress [86]; Gcn4p, Msn2p and Msn4p during amino acid starvation [86]; Hsf1p during variable heat shock elements including hyperthermia [86]; Sfp1p during DNA damage [84]; and Spt23p and Mga2p during cooling [87]. The stress conditions are associated with invasive growth (regulated by Tec1p, Ste12p, Ash1p and Sok2p), and with the need for chromatin remodeling (regulated by Snf6p, Snf2p, Spt20p, Tup1p and Swi3p) and DNA repair (regulated for instance by Abf1p and Spt10p) [84,86].

#### Coherence

**Figure 25:**
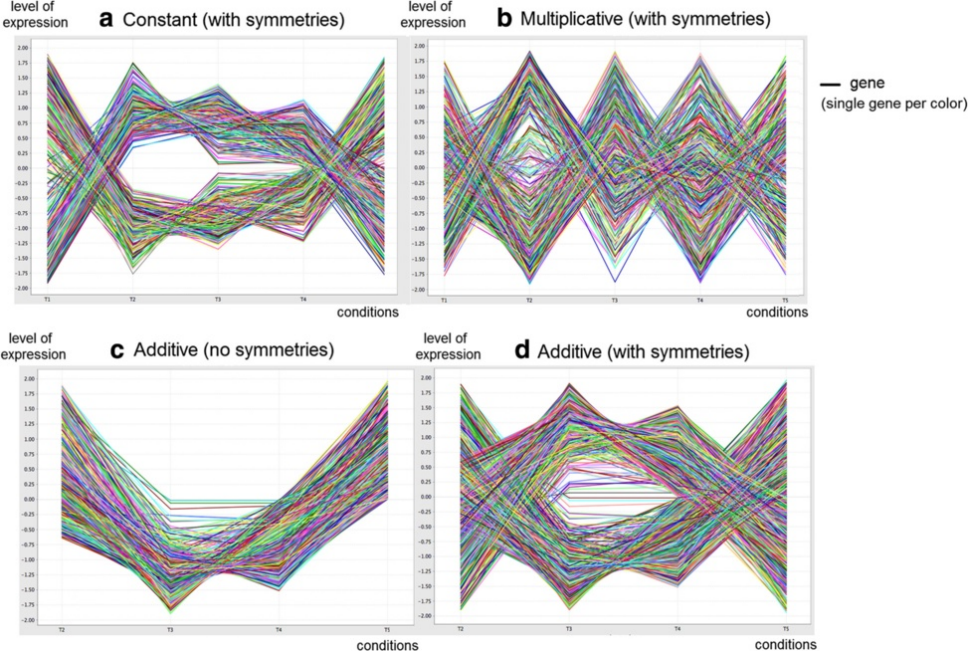
**Biclusters extracted from gasch dataset with constant models (a), multiplicative models (b) and additive models in the absence and presence of symmetries (c and d).**

Figure 25 illustrates four biclusters discovered in the *gasch* dataset, which are related with the response of Yeast genes to heat shock at different time points. BicPAM’s behavior is particularly favorable to the discovery of these biclusters, contrasting with other biclustering approaches. In particular, the combination of constant models with symmetries, multiplicative models with symmetries, additive models with several levels of expression, and additive models with symmetries. The analysis of these biclusters shows the relevance of combining multiple levels of expression ($|\mathcal {L}|\geq 5$) with noise relaxations for the discovery of meaningful biclusters. Additionally, this analysis supports the importance of allowing sign-changes across multiple levels of expression to capture activation and repression mechanisms in regulatory processes.

### Comparison of pattern-based biclustering approaches

In the previous sections, we provided substantial empirical evidence for the improvements of BicPAM performance in comparison with peer pattern-based methods such as BiModule, DeBi and RAP. First, Figures 10 and 11 show the unique ability of BicPAM to discover non-constant biclusters (>50 percentage points in MS and FC against BiModule, DeBi and RAP). Second, Figure 12 shows improvements in the discovery of constant biclusters related with BicPAM’s ability to deal with the items-boundary problem and to adequately postprocess biclustering solutions. Additionally, BicPAM’s ability to combine solutions discovered under multiple levels of expression and to discover all the maximal biclusters (closed pattern representations) surpasses specific drawbacks found in some of the existing methods. Third, the incorporation of scalability principles and of minimalist FP-trees (Figure 20) guarantee its competitive computational complexity even when procedures to handle noise and adapt the biclustering structures are used. Fourth, Figures 22 to 24 show significant performance improvements of BicPAM due to its exclusive ability to deal with medium-to-high levels of missing values and noise. Finally, the biological relevance of BicPAM’s solutions against the solutions provided by the peer methods is assessed in Table 4 and further supported in subsequent analyzes. In particular, we show that BicPAM’s solutions cover the (enriched) biological processes associated with peer pattern-based solutions (Table 6). Moreover, they enable the discovery of unique and biologically meaningful biclusters (Tables 5 and 6) such as the four illustrative biclusters in Figure 25.

## Conclusion

A new approach for flexible and robust pattern-based biclustering (BicPAM) is proposed with the goal of performing exhaustive searches to discover biclustering solutions with multiple coherencies under relaxed conditions (arbitrary number and structure of biclusters) with heightened efficiency. BicPAM is the result of integrating existing dispersed contributions on pattern-based biclustering with new critical methods to deal with more flexible expression profiles and to handle varying levels of missing values and noise.

BicPAM goes beyond the constant assumption made by existing pattern-based approaches, and extends the biclustering task to new types of biclusters, including additive and multiplicative assumptions that can accommodate symmetries. It is the first attempt to model these coherencies under a pattern-based approach. This is critical since pattern-based searches are exhaustive, support flexible structures of biclusters, and consider multiple levels of expression (instead of differential expression).

Additionally, BicPAM is able to surpass the common drawbacks related with discretization procedures, since it is able to assign multiple items over a single element to tackle the items-boundary problem. In this way, the transactional database derived from the input matrix can have more items than the number of elements in the original matrix.

BicPAM relies on dynamic parameterizations for a tuned performance across different settings, including pattern representations, strategies to handle missing values, and postprocessing options for the post-handling of noise and composition of flexible structures. Although the default options are dynamically derived based on the properties of the target dataset, they can also be defined by the user without the need to adapt the core mining task.

Results on both synthetic and real datasets show BicPAM’s ability to find optimal solutions over matrices with more than 10.000 rows and up to 400 columns. The assessment of BicPAM’s performance against peer pattern-based approaches and other state-of-the-art biclustering algorithms supports its heightened flexibility and robustness to noise. Additionally, we observed that the majority of the biclusters discovered by BicPAM in gene expression datasets are functionally relevant and could not be discovered by other biclustering approaches. The analysis of their transcriptional regulation showed significant and meaningful associations.

## Software availability

The datasets and BicPAM executables are available in http://web.ist.utl.pt/rmch/software/bicpam/.

## Endnote

^a^ Clustering metrics measure the ability to correctly group rows (or columns), that is, of attaining high intra-cluster similarity and low inter-cluster similarity. Entropy and F-measure metrics are the common choice [56,57]. F-measure can be further decomposed in terms of recall (coverage of found rows by a hidden cluster) and precision (absence of rows present in other hidden clusters).
